# Splicing of branchpoint-distant exons is promoted by Cactin, Tls1 and the ubiquitin-fold-activated Sde2

**DOI:** 10.1093/nar/gkac769

**Published:** 2022-09-12

**Authors:** Anupa T Anil, Karan Choudhary, Rakesh Pandian, Praver Gupta, Poonam Thakran, Arashdeep Singh, Monika Sharma, Shravan Kumar Mishra

**Affiliations:** Department of Biological Sciences, Indian Institute of Science Education and Research (IISER) Mohali, Sector 81, 140306 Punjab, India; Department of Biological Sciences, Indian Institute of Science Education and Research (IISER) Mohali, Sector 81, 140306 Punjab, India; Department of Biological Sciences, Indian Institute of Science Education and Research (IISER) Mohali, Sector 81, 140306 Punjab, India; Department of Biological Sciences, Indian Institute of Science Education and Research (IISER) Mohali, Sector 81, 140306 Punjab, India; Department of Biological Sciences, Indian Institute of Science Education and Research (IISER) Mohali, Sector 81, 140306 Punjab, India; Department of Biological Sciences, Indian Institute of Science Education and Research (IISER) Mohali, Sector 81, 140306 Punjab, India; Department of Chemical Sciences, Indian Institute of Science Education and Research (IISER) Mohali, Sector 81, 140306 Punjab, India; Department of Biological Sciences, Indian Institute of Science Education and Research (IISER) Mohali, Sector 81, 140306 Punjab, India

## Abstract

Intron diversity facilitates regulated gene expression and alternative splicing. Spliceosomes excise introns after recognizing their splicing signals: the 5′-splice site (5′ss), branchpoint (BP) and 3′-splice site (3′ss). The latter two signals are recognized by U2 small nuclear ribonucleoprotein (snRNP) and its accessory factors (U2AFs), but longer spacings between them result in weaker splicing. Here, we show that excision of introns with a BP-distant 3′ss (e.g. *rap1* intron 2) requires the ubiquitin-fold-activated splicing regulator Sde2 in *Schizosaccharomyces pombe*. By monitoring splicing-specific *ura4* reporters in a collection of *S. pombe* mutants, Cay1 and Tls1 were identified as additional regulators of this process. The role of Sde2, Cay1 and Tls1 was further confirmed by increasing BP–3′ss spacings in a canonical *tho5* intron. We also examined BP-distant exons spliced independently of these factors and observed that RNA secondary structures possibly bridged the gap between the two signals. These proteins may guide the 3′ss towards the spliceosome's catalytic centre by folding the RNA between the BP and 3′ss. Orthologues of Sde2, Cay1 and Tls1, although missing in the intron-poor *Saccharomyces cerevisiae*, are present in intron-rich eukaryotes, including humans. This type of intron-specific pre-mRNA splicing appears to have evolved for regulated gene expression and alternative splicing of key heterochromatin factors.

## INTRODUCTION

Splicing of precursor mRNAs into protein-coding mRNAs is an essential step in gene expression. It also promotes regulated gene expression and alternative splicing. The process is completed by the spliceosome assembled from five small nuclear RNA-protein (snRNP) complexes. Spliceosomes excise pre-mRNA introns detected by their splicing signals. The 5′-splice site (5′ss) is detected by U1 snRNP, while the branchpoint (BP), the polypyrimidine tract and the 3′-splice site (3′ss) are detected by U2 snRNP and its accessory factors (U2AFs) (1,2). Further, the U4/U6–U5 tri-snRNPs are recruited to form the B complex spliceosome (3,4). The unwinding of U4/U6 snRNAs by Brr2 helicase mediates the formation of the U2/U6 snRNP complex. The active site embedded within the U2/U6–U5 snRNP complex is formed with two catalytic metal-binding sites in U6 snRNA, which catalyses both transesterification reactions needed to excise the intron and ligate the two exons (5–8).

Introns differ in positions and sequences of splicing signals, lengths and location in the pre-mRNA. Furthermore, the presence of sequence motifs that act as splicing enhancers or silencers, and the propensity to form secondary structures, also differ (9–13). This diversity is critical for regulated gene expression and alternative splicing; therefore, intron-rich eukaryotes experience an enormous impact of RNA splicing on their physiology. To tackle the processing of diverse introns, intron-specific splicing regulators that include RNP assembly factors, RNA-binding proteins or RNA- and protein-modifying proteins and enzymes are recruited to the spliceosome (14).

Ubiquitin and ubiquitin-like proteins (e.g. SUMO, Hub1 and Sde2; collectively referred to as UBLs) are post-translational modifiers of proteins controlling diverse cellular activities. They also regulate pre-mRNA splicing by modifying spliceosomes, thereby potentiating the machinery to act on specific introns and pre-mRNAs (15). UBLs appear dispensable for constitutive pre-mRNA splicing involving introns with canonical splicing signals, but become critical for excising introns with non-canonical sites. For example, the UBL Hub1/UBL5 is conserved from *Saccharomyces cerevisiae* to humans. It promotes alternative splicing and facilitates spliceosomal recognition of suboptimal 5′ss. It modifies spliceosomes by binding to HIND-containing splicing factors (16–19). Hub1 from *S. cerevisiae* also activates the spliceosomal RNA helicase Prp5, allowing Hub1-modified spliceosomes to use a non-canonical 5′ss and allow alternative splicing (19).

Spliceosomes modified by the ubiquitin-fold-activated Sde2 promote the excision of selected introns in a subset of pre-mRNAs in *Schizosaccharomyces pombe*. This splicing regulator is conserved among intron-rich eukaryotes up to humans, but is absent in intron-poor organisms such as *S. cerevisiae*. Sde2 was recently shown to be important in pre-mRNA splicing in mammalian cells (20). Sde2 is translated as an inactive precursor harbouring an N-terminal UBL (Sde2_UBL_) that is processed by deubiquitinating enzymes (DUBs) in a conserved GG–K motif to form activated ^K^Sde2-C (21,22). Following processing by DUB, Ubp5 and Ubp15 in *S. pombe*, Sde2 matures into spliceosomal-competent ^K^Sde2-C with lysine at the N-terminus. The removal of Sde2_UBL_ and the free lysine of ^K^Sde2-C are critical for its intron-specific splicing function. However, the question of why only selected pre-mRNAs required Sde2 for efficient splicing remained unanswered.

Here, we report that Sde2 facilitates excision of introns with longer spacing between the BP and 3′ss (referred to as BP-distant 3′ss). This activity requires the cleavage of Sde2_UBL_ by DUB and the free lysine of processed ^K^Sde2-C. Using splicing reporters made with the *ura4* gene split by introns of varying distances between the BP and 3′ss, we searched for *S. pombe* deletion mutants of putative splicing factors. We identified Cactin/Cay1 and Tls1 as additional regulators of BP-distant splicing. These three splicing regulators are absent in budding yeast but are conserved from the fission yeast *S. pombe* to metazoans. Furthermore, by altering the spacing between the BP and 3′ss in selected targets, we show that introns with longer spacing between the BP and 3′ss require Sde2, Cay1 and Tls1 for efficient splicing. They regulate gene expression and alternative splicing of various heterochromatin factors, including the telomeric factor Rap1.

## MATERIALS AND METHODS

### Plasmids, strains, *S. pombe* transformation and growth assays

Plasmids and strains used in this study are listed in Supplementary Tables S2 and S3, respectively. *The S. pombe* deletion strains of splicing factors were obtained from the Bioneer haploid deletion library. Competent cell preparation and transformation were performed following previously published protocols (23,24). Double knockout strains for genetic interactions were made by tetrad dissection of spores. The plasmid clones indicated in Supplementary Figure S12A were used to tag genes at their chromosomal loci. The NotI inserts were transformed into wild-type (wt) *S. pombe* strains, and tagging was confirmed by western blot analysis. Polymerase chan reaction (PCR) and western blotting confirmed gene deletions and tagging, respectively (23,24). The reporter strain for telomeric silencing in *Δsde2* (25) was obtained from NBRP-yeast, Japan. Plasmids expressing chromatin factors had the *nmt81* promoter inducible in the absence of thiamine and the *S. cerevisiae tef2* terminator. Overexpression was carried out by expressing clones under a strong *eno101* promoter and a *tef2* terminator. For growth/spot assays, 5-fold serial dilutions of cells were spotted on the indicated agar plates, and plates were incubated at temperatures indicated in the figure legends.

### Splicing reporters

The reporters used in this study are listed in Supplementary Table S4. Splicing reporter plasmids have the *S. pombe ars1*, *leu2+* selection marker and *ura4* cDNA with introns inserted between nucleotides 426 and 427. Insertions at this position in *ura4* were previously used to study exon skipping (26). The reporters have a 400 bp segment of the *S. pombe eno101* promoter, an N-terminal 3MYC tag and the *tef2* terminator. Intronic variants were inserted into the *ura4* reporter using specific primers in SOE (splicing by overlap extension) PCR. Point mutations in the BP and 3′ss were made by QuikChange site-directed mutagenesis (Agilent). For reporter assays and screens, competent cells from *S. pombe* strains were transformed with the reporter and transformants were selected in media lacking leucine. Five-fold serial dilutions of cells were spotted on media lacking uracil, and counter-selection was performed on media containing 0.1% 5-fluoroorotic acid (5-FOA). The plates were incubated for 3–5 days at 30°C.

### Bioinformatics of *S. pombe* introns

The Sde2-dependent introns were obtained from the splicing-sensitive microarray of the *Δsde2* strain (GEO accession number GSE97097) (22). Nucleotide sequences were obtained from the PomBase server. RNA structures were predicted on the RNAfold WebServer (27). For intron analysis, the complete dataset of introns was downloaded from PomBase and then mapped with the microarray data. A cut-off of 0.5 log_2_*Δsde2/*wt ratio (∼1.4-fold difference) of intron-retention values obtained with the strains grown at 30°C was applied. Introns with ratios >0.5 were considered Sde2 dependent, and the remaining introns were considered Sde2 independent. The database was then matched with the following list of BP sequences: ‘CCAAC’, ‘CUAAC’, ‘UUAAC’, ‘UCAAC’, ‘CCGAC’, ‘CUGAC’, ‘UUGAC’, ‘UCGAC’,‘CCAAU’, ‘CUAAU’, ‘UUAAU’, ‘UCAAU’, ‘CCGAU’, ‘CUGAU’, ‘UUGAU’, ‘UCGAU’, ‘GUAAC’, ‘CUCAC’, ‘AUAAC’, ‘UUGAC’, ‘CUGAU’, ‘CUAAA’, ‘CUUAC’ and ‘CUAAG’. For both Sde2-dependent and -independent introns, the distances of BP sequence (closest to the 3′ss ‘NAG’) from the 5′ and 3′ splice sites were calculated by counting the number of bases in between, and frequency distributions were computed for comparative analysis. Polypyrimidine tracts were defined as at least six consecutive non-adenine nucleotides containing no fewer than three uridines (9) and were identified using an in-house-written script. For sequence logos, the nucleotides spanning the desired window around the 5′ss, BP nucleotides and 3′ss for Sde2-dependent and -independent introns were obtained using custom scripts in R. Sequence logos were then created by using the ggseqlogo package in R (28). Violin plots were generated using the ggplot2 package in R (https://link.springer.com/book/10.1007/978-0-387-98141-3).

### RNA isolation and cDNA synthesis

For RNA isolation, logarithmically growing cells equivalent to 2 OD_600_ were harvested (at OD_600_ ∼0.5–0.6). The hot acid–phenol method was used for RNA isolation, and 2 ml phase-lock tubes (Qiagen) were used for phase separation (29). Multiscribe reverse transcriptase with random hexamer primers (Thermofischer Scientific) was used to synthesize cDNAs from 1 μg of total RNA incubated at 42°C for 16 h. Splicing defects were monitored by detecting intron-containing transcripts or transcripts matured post-splicing. Reporter-specific intron-containing transcripts were detected using a cDNA template by PCR with a 3MYC tag-specific primer and *a ura4* exon 2-specific primer. The reporter-specific transcripts matured post-splicing were detected using the 3MYC tag-specific primer and the exon–exon junction-specific primer (where the first half of the primer annealed to *ura4* exon 1 and the second half of the primer annealed to exon 2). The PCR products were run on a 2% agarose gel. Real-time quantitative PCR (qRT-PCR) was performed using SYBR green (Roche). The primers used for the splicing assay and qRT-PCR are listed in Supplementary Table S5. Lariat PCR was described in (30). Briefly, 2 μg of DNase I-treated RNA was heat denatured at 65°C for 5 min, and cDNA synthesis was performed in the presence of 3 mM MgCl_2_ and 3 mM MnCl_2_ for 2 h at 37°C with a reverse primer specific for the target introns.

### Western blots

For protein western blots (immunoblotting), logarithmically growing cells equivalent to 2 OD_600_ were harvested. Whole protein was extracted using the trichloroacetic acid (TCA) method (24). Total proteins used for western blots in Supplementary Figure S12B were isolated by heating cells in high urea buffer at 65°C for 15 min. The protein samples were run on sodium dodecylsulphate–polyacrylamide gel electrophoresis (SDS–PAGE) gels, transferred to a polyvinylidene difluoride (PVDF) membrane and probed with specific primary and secondary antibodies.

### Antibodies

Anti-MYC antibodies raised in rabbits (MYC polyclonal), anti-haemagglutinin (HA) raised in mice (HA, monoclonal), anti-mouse horseradish peroxidase (HRP; goat) and anti-rabbit HRP (goat) were procured from Sigma-Aldrich.

## RESULTS

### Intronic features define the role of Sde2 in pre-mRNA splicing

**Figure 1.**
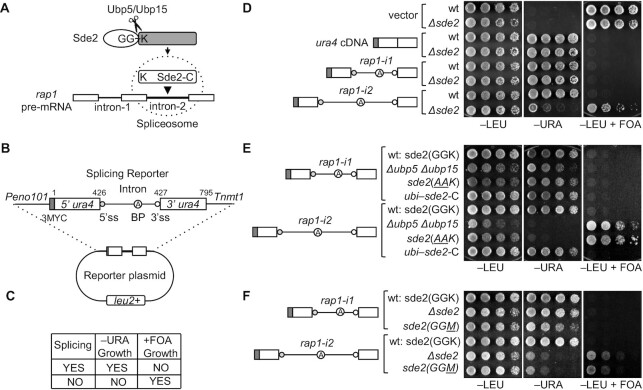
Intronic features define the role of Sde2 in pre-mRNA splicing. (**A**) Schematic of Sde2 activation and function for the splicing of *rap1-i2*. (**B**) Design of splicing reporters (ss, splice site; BP, branchpoint). The numbers on the reporter show the insertion site of introns in the *ura4* gene. *rap1-i1* and *i2* were individually inserted at the designated insertion site mentioned in the schematic to generate *rap1-i1* and *rap1-i2* reporters, respectively (**C**) Table showing the expected result from the reporter in *S. pombe*. (**D**) The splicing-proficient strain grows on –URA plate but does not grow on +FOA plates, and vice versa (incubation time: –LEU, –URA, 4 days; +FOA, 5 days). (**E**) Ubiquitin-like processing of Sde2 is essential for its intron-specific splicing activity. The Sde2 processing-defective strains *Δubp5 Δubp15* and *sde2 (AAK)* could not splice *the rap1-i2* reporter, while the ubiquitin–^K^Sde2-C chimeric strain (*Ubi*–*sde2-C*) spliced the reporter (incubation time: –LEU, –URA, 4 days; +FOA, 5 days). (**F**) The N-terminal lysine of ^K^Sde2-C is crucial for the intron-specific splicing activity of Sde2. The *sde2* (*GGM*) mutant strain was unable to splice *rap1-i2* (incubation time: –LEU, –URA, 4 days; +FOA, 4 days).

We have previously reported that *S. pombe* Sde2 is a ubiquitin-fold-activated splicing regulator that facilitates pre-mRNA splicing in an intron-specific manner (22). Splicing-sensitive microarrays were performed with the wt and the *Δsde2* strain to understand the role of Sde2 in pre-mRNA splicing. The microarray data, further verified by RT–PCR, showed that the lack of Sde2 (*Δsde2*) leads to the retention of only selected introns (22). A key splicing target of Sde2 is *rap1* pre-mRNA encoding the telomere-binding shelterin complex subunit Rap1 (Figure 1A; Supplementary Figure S1A, B). However, it is unknown why only specific introns, such as intron 2 but not intron 1 of *rap1*, required Sde2 for splicing.

To understand the intron specificity of Sde2, we made two splicing reporters with the *S. pombe ura4* gene split individually by the two *rap1* introns (Figure 1B). We tested the reporters' ability to complement a *ura4* auxotrophic *S. pombe* strain. Functional *ura4* mRNA and protein were expected only after accurate splicing of the pre-mRNAs that would allow the cells to grow on plates lacking uracil (−URA) but not on uracil counter-selection plates containing 5′-FOA (+FOA) (Figure 1C). With the reporter split by the Sde2-independent *rap1* intron 1 (*rap1-i1* reporter), both the wt and *Δsde2* strains grew on −URA plates but not on +FOA. In contrast, with the reporter split by the Sde2-dependent *rap1* intron 2 (*rap1-i2* reporter), wt cells grew on −URA but not on the counter-selection plate containing FOA, while *Δsde2* cells did not grow well on −URA but grew on the +FOA plate (Figure 1D).

The above results suggest that *rap1* intron 2 (but not intron 1) requires Sde2 for splicing. The intron specificity was further confirmed by detecting reporter-specific proteins in western blots and transcripts in reverse transcription–PCR (RT–PCR) assays. For these assays, a DNA sequence encoding the 3MYC epitope tag was inserted at the N-terminus of the reporters (Figure 1B). In RT–PCR, one primer was annealed to 3MYC and another to *ura4* exon 2 or the junction of the two exons. The *rap1-i1* reporter was spliced in both wt and *Δsde2* strains. In contrast, the *rap1-i2* reporter was spliced only in the wt but not in *Δsde2* (Supplementary Figure S1C, D). The binding specificity of the junction primers for the *ura4* cDNA was verified by performing multiplex PCR on plasmid DNA with and without the intron as templates (Supplementary Figure S1E). Anti-MYC western blots were used to detect reporter-specific proteins. The *rap1-i1* reporter formed the full-length Ura4 protein in both wt and *Δsde2* strains, whereas the *rap1-i2* reporter formed the full-length Ura4 protein only in the wt but not in *Δsde2*. In general, both reporters were partially spliced. They showed truncated Ura4-N proteins resulting from intron retention in all strains, indicating that both *rap1* introns were weak once kept out of their native location (Supplementary Figure S1F). However, *rap1* intron 2 needed Sde2 for splicing. Thus, the splicing role of Sde2 was attributed to specific features present in the intron. These assays also ruled out the possible involvement of transcription, untranslated regions (UTRs) or the protein-coding parts of the *rap1* gene for its Sde2 dependency (see the next section).

Sde2 precursors in *S. pombe* and humans are processed into Sde2_UBL_ and ^K^Sde2-C (21,22). After the DUBs Ubp5 and Ubp15 cleave the *S. pombe* precursor at the conserved GG–K motif, the processed ^K^Sde2-C enters the spliceosome (Figure 1A). ^K^Sde2-C is short-lived due to its proteasomal degradation by the N-end rule pathway (22,31). A ubiquitin–^K^Sde2-C chimaera complemented growth defects in the *Δsde2* strain, suggesting that ubiquitin could replace the Sde2_UBL_ activity of producing functional ^K^Sde2-C. We tested the importance of ubiquitin-like processing for reporter splicing in three strains: (i) a DUB deletion strain *Δubp5 Δubp15;* (ii) a processing-defective *sde2 (AAK)* mutant (amino acid changes underlined); and (iii) a ubiquitin–^K^Sde2-C chimaera that replaces chromosomal *sde2* (Figure 1E; Supplementary Figure S1G, H). Like the *Δsde2* strain, the *sde2* processing-defective strains *Δubp5 Δubp15* and *sde2 (AAK)* spliced only the *rap1-i1* reporter and became uracil positive. However, neither of the strains could splice the *rap1-i2* reporter and remain uracil negative. In contrast, the Sde2-replacing ubiquitin–Sde2-C chimeric strain spliced both reporters and became uracil positive (Figure 1E). We also tested the need for N-terminal lysine in ^K^Sde2-C in a lysine to methionine mutant strain *sde2 (GGM)*. This mutant, which forms ^M^Sde2-C, could splice only the *rap1-i1* reporter but not *rap1-i2* (Figure 1F). Thus, both ubiquitin-like processing and the N-terminal lysine of ^K^Sde2-C are essential for the *rap1* intron 2-specific splicing activity of Sde2.

### Introns with longer spacing between BP and 3′ss require Sde2 for splicing

Next, we ask which features of the intron targets detected in the microarrays made them dependent on Sde2. The target introns (defined as log_2_*Δsde2/*wt signal ratio at 30°C ≥0.5) lacked a common sequence motif and did not match with respect to the strengths of their splicing signals, lengths or position in resident pre-mRNAs. The 5′ss in the Sde2 target introns showed a reduced enrichment of the nucleotide ‘A’ at positions +3 and +4 compared with Sde2 non-targets. Although most targets did not have a weak BP or 3′ss, a few had a non-canonical BP and 3′ss or even their combinations (Supplementary Table S1). The Sde2-dependent introns had an average of 3.32 polypyrimidine tracts between the 5′ss and BP (compared with 2.40 in non-targets) and 0.38 between the BP and 3′ss (0.25 in non-targets) (Supplementary Figure S2A, B). For testing whether Sde2 dependency was due to a non-canonical 5′ss, the 5′ss of *rap1* intron 2 was changed to a canonical site (GTATGA to GTAAGT) in the genomic construct of *rap1*. RT–PCR was used to study splicing efficiency in wt and *Δsde2* strains (Supplementary Figure S3A). We did not observe an improvement in its splicing in the *Δsde2* strain. We also made reporters with non-canonical variants of the BP and 3′ss in an Sde2-independent *tho5* intron 1 (*tho5-i1*) [Tho5 is a THO complex subunit (32)] and tested their splicing in wt and *Δsde2* strains. All the BP and 3′ss variant reporters allowed a similar growth of wt and *Δsde2* strains on −URA plates, suggesting their similar usage and Sde2 independence. When monitored by growth on +FOA plates, reporters with BP variants UUAAC and CUAAA (CUAAC = canonical BP) and 3′ss variant AAG (UAG = canonical 3′ss) showed modest growth of *Δsde2*, suggesting a suboptimal use of weak BPs and 3′ss in this mutant (Supplementary Figure S3B, C). Subtle splicing defects of BP and 3′ss variants could not explain their Sde2 dependency. A combination of weak splicing signals could enhance the Sde2 dependency of an intron.

**Figure 2.**
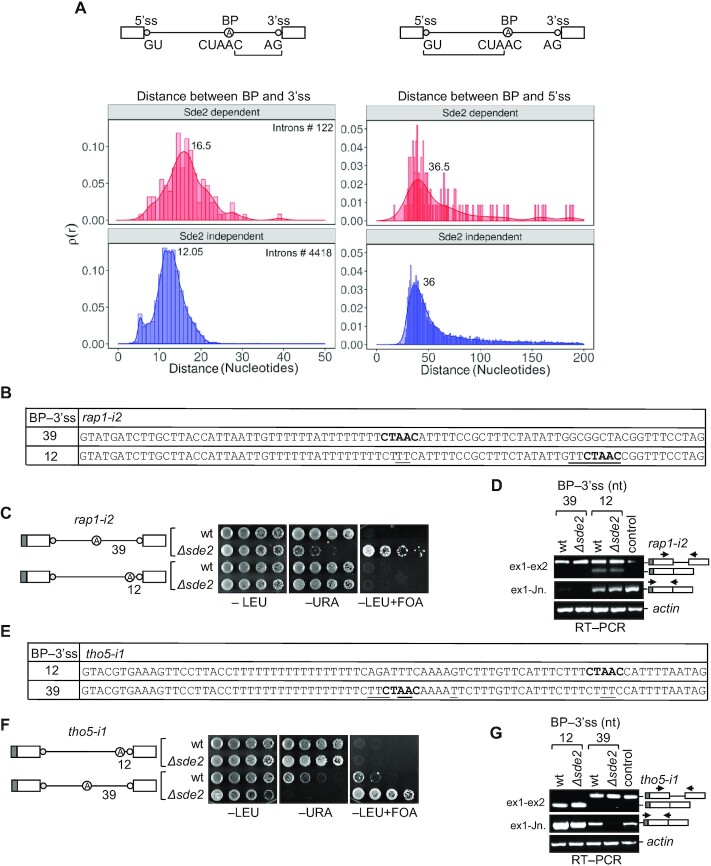
Sde2 target introns have longer spacing between the BP and 3′ss. (**A**) Distribution of distances (number of nucleotides) between the BP adenosine and the 5′- and 3′-splice sites (ss) of the Sde2-dependent and -independent introns (Sde2-dependent ≥0.5 log_2_*Δsde2/*wt ratio at 30°C; Sde2-independent ≤log_2_*Δsde2/*wt ratio at 30°C). Red peaks and histograms show 122 Sde2-dependent introns, and blue peaks and histograms show 4418 Sde2-independent introns. The numbers on the peaks show their maxima. (**B**) Nucleotide sequence of *rap1-i2* with different spacing between the BP and 3′ss. (**C** and **D**) Growth assay and RT–PCR showing that *rap1-i2* with 39 nt between the BP and 3′ss was Sde2 dependent (incubation time: –LEU, –URA 4 days; +FOA 5 days). The reduction of this spacing made its splicing Sde2 independent. RT–PCR was performed by two sets of primers as indicated in the schematic. ex1-ex2 indicates PCR performed with primers that bind to exon 1 and exon 2, thereby monitoring both spliced and unspliced *ura4* transcripts, and ex1-Jn indicates PCR performed with primers that anneal to exon 1 and the exon 1–exon 2 junction, thus monitoring only the spliced *ura4* transcripts. The arrows in the schematics depict the binding of primers in the reporter. (**E**) Nucleotide sequence of *tho5-i1* with different spacing between the BP and 3′ss. (**F** and **G**) Growth assay and RT–PCR showing that an Sde2-independent intron, *tho5-i1*, required Sde2 for splicing after increasing the distance between the BP and 3′ss. Growth assay and RT–PCR were performed similarly to (C) and (D) (incubation time: –LEU, –URA 4 days; +FOA, 5 days). Arrows in the schematics depict primer binding to the reporter.

After further scrutiny of the Sde2 target introns, we noticed that the spacing between the BP and 3′ss in most of them was longer than the average spacing of introns in *S. pombe* (Figure 2A; Supplementary Figure S2C). The spacing is defined as the RNA bases between branch adenosine and the 3′ss. The BP and 3′ss in *S. pombe* introns are on average 12 nt apart (9). For example, the BP and 3′ss are 39 nt apart in *rap1* intron 2 but only 14 nt apart in *rap1* intron 1. We tested the importance of a longer BP–3′ss spacing in the *rap1-i2* reporter by bringing its BP closer at 12 nt to the 3′ss (Figure 2B–D). Interestingly, this intron was excised in the*Δsde2* strain, as monitored by the growth on −URA plates and lack of growth on +FOA plates. In contrast, the spacing between the 5′ss and BP in *rap1* intron 2 was not responsible for its Sde2 dependency, as increasing this distance did not rescue the splicing defect in *Δsde2* (Supplementary Figure S4A). We made reporters with two more target introns, *ftp105* intron 3 and *pyp3* intron 1, and observed a similar Sde2 dependency for both. [The Ftp105 protein functions at the Golgi (33), and Pyp3 encodes a protein tyrosine phosphatase (34).] To verify if the longer spacings between the BP and 3′ss made them Sde2 dependent, we reduced the gap in *ftp105* intron 3 from 24 nt to 12 nt. Sde2 was not needed for the excision of this intron variant (Supplementary Figure S4B, C).

**Figure 3.**
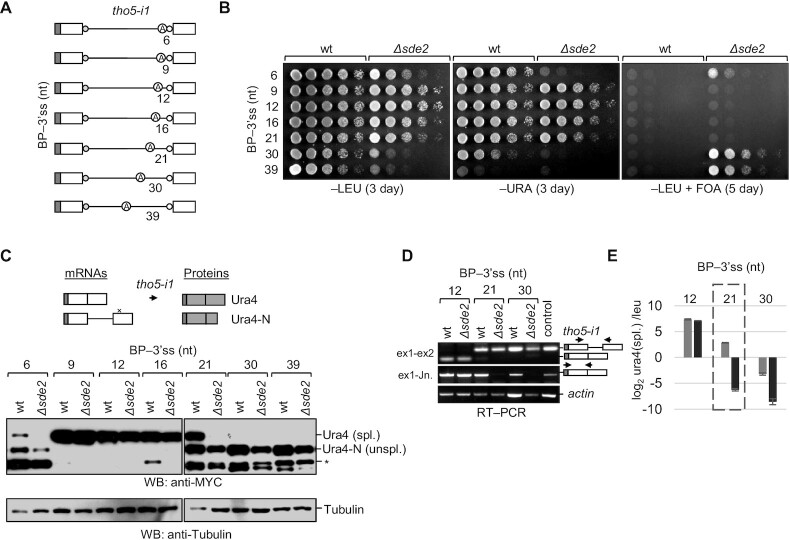
Threshold distance between the BP and 3′ss for the Sde2 target introns. (**A**) Schematics of *tho5-i1* reporters with increasing spacing between the BP and 3′ss. (**B, C**) Growth assay and immunoblot analysis show that an increase in the distance between the BP and 3′ss makes the intron Sde2 dependent (incubation time: –LEU, –URA, 3 days; +FOA, 5 days). × marks the stop codon that arises during the translation of intron-retained mRNA. * marks proteins possibly arising from aberrant splicing or proteolytic cleavage. (**D**) Semi-quantitative RT–PCR showing splicing of *tho5-i1* reporters with different spacing between the BP and 3′ss. RT–PCR was performed in the same way as in Figure 2D. Small amounts of Ura4 protein appeared enough for the cells to grow on –URA, but the cDNA and protein's detection by RT–PCR and immunoblots seems to require more efficient splicing. Thus, growth assays discriminate the reporters’ activities better with lower splicing efficiency, whereas RT–PCR and immunoblots discriminate the reporters’ activities better with higher splicing efficiency. (**E**) qRT-PCR analysis to quantify spliced transcripts from the *tho5-i1* reporter with different spacings between the BP and 3′ss in wt (grey bars) and *Δsde2* (black bars). The quantitation was against *leu2* transcripts arising from the same plasmid. The forward primer used in qRT-PCRs binds to exon 1, and the reverse primer binds to the exon 1–exon 2 junction, thus specifically amplifying the spliced transcripts. The *y*-axis denotes log_2_spliced ura4 transcripts against leucine transcripts, and the *x*-axis depicts different spacings between the BP and 3′ss in the *tho5-i1* reporter.

In contrast, an Sde2-independent *tho5* intron 1, with 12 nt between the BP and 3′ss, became Sde2 dependent when its BP was moved farther to 39 nt from the 3′ss (Figure 2E–G). Since this variant was poorly spliced even in wt cells, indicated by the weak growth on −URA plates, we gradually moved the BP away from the 3′ss to find the BP–3′ss distance threshold for efficient splicing (Figure 3A). Reporters with 9, 12, 16 and 21 nt spacing allowed growth on −URA plate of both the wt and *Δsde2* strain, while the reporters with 6 and 30 nt spacing allowed the growth of the wt but not *Δsde2* (Figure 3B). In immunoblots, 9 and 12 nt spacing of reporters gave a maximum amount of Ura4 protein, but the protein level decreased rapidly after increasing the BP–3′ss spacing. The intron with 21 nt spacing showed the most striking difference in splicing efficiency between the wt and *Δsde2*. This result indicated that Sde2-C becomes critical for using the 3′ss at ≥21 nt from the BP (Figure 3C). Interestingly, the intron with a 6 nt gap between the BP and 3′ss showed reduced levels of Ura4 protein on immunoblot. The defect might be due to steric clashes in the spliceosome when the BP and 3′ss are too close. The results were further validated by RT–PCR and qRT-PCR assays for selected reporters (Figure 3D, E; Supplementary Figure S4D). Furthermore, there is a limit to the spacing between the BP and 3′ss for efficient splicing. Splicing efficiency decreased drastically when we further increased BP–3′ss spacing, and introns with ≥30 nt spacing between the BP and 3′ss were poorly excised even in wt cells (Figure 3B, C). The correct usage of the newly introduced BP was verified by sequencing PCR products from lariats in the *rap1-i2* distance-reduced reporter (BP to 3′ss = 12 nt) and a *tho5-i1* reporter variant (BP to 3′ss = 30 nt) (Supplementary Figure S5A, B).

**Figure 4.**
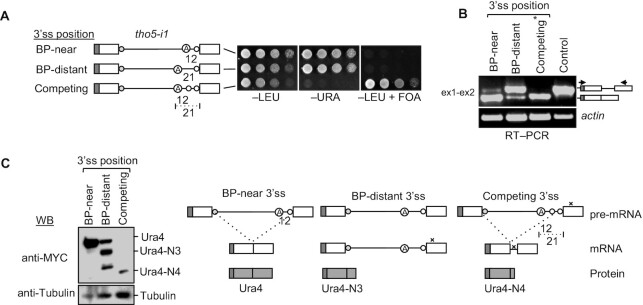
The spliceosome prefers BP-near 3′ss over BP-distant 3′ss. (**A**) Splicing of *tho5-i1* reporters with competing 3′ss (BP-near and BP-distant) in the wt was tested on –URA and +FOA plates (incubation time: –LEU, –URA, 3 days; +FOA, 4 days). (**B**) Semi-quantitative RT–PCR monitoring splicing of BP-near and BP-distant 3′ss in the *tho5-i1* reporter. Primers are as shown in the block diagram (ex-exon). * marks the band that appears due to the use of BP-near 3′ss, which leads to the incorporation of nine additional nucleotides in the *ura4* cDNA transcript. (**C**) Western blot analysis shows the translational product of mRNA spliced using BP-near 3′ss but not from BP-distant 3′ss. Ura4-N3 and -N4 indicate proteins from mRNAs with exon 1 up to the stop codon (labelled with x).

We next tested whether Sde2 could enhance splicing through a BP-distant 3′ss in the presence of a BP-near 3′ss. For this, we made a reporter with two competing 3′ss, BP-near and BP-distant, and monitored its splicing in wt cells (Figure 4A–C). A protein corresponding to the mRNA formed after the usage of only the BP-near 3′ss could be detected, suggesting that BP-distant splicing is avoided in the presence of a more favourable BP-near 3′ss.

### RNA between BP-distant 3′ss could be structured

Among ∼5250 introns in *S. pombe*, the spacing of BP–3′ss in ∼40 introns is ≥30 nt. The spacing is ≥20 nt in ∼90 introns. Despite a longer spacing between the BP and 3′ss, several introns did not appear to require Sde2 (22). Thus, the question arose of how those introns were excised in *Δsde2*. One possibility was that the sequence between the BP and 3′ss of these introns might form secondary structures, thus bringing their 3′ss closer to the BP. BP–3′ss intervening segments in several introns were predicted to fold into secondary structures with shorter effective distances between the BP and 3′ss. Such secondary structures were detected in, for example, intron 1 of *cam1* [encoding calmodulin (35)], intron 1 of *ste4* [encoding MAPK cascade adaptor protein (36)], intron 2 of *fmd1* [encoding formaldehyde dehydrogenase (37)], intron 1 of *whi5* [encoding a transcription repressor (38)], intron 2 of *atg20* [encoding an autophagy protein (39)] and intron 2 of *mug65* [encoding a dysferlin-like membrane trafficking factor (40)] (Supplementary Figures S6 and S7A). In such cases, the disruption of secondary structures by exposure to elevated temperatures could open the fold and increase the distance between the BP and 3′ss. We examined this possibility by RT–PCR assays. Indeed, excision of introns with secondary structures such as *rap1* intron 2, *whi5* intron 1, *atg20* intron 2 and *mug65* intron 2 was sensitive to 15 min treatment at 42°C. After lowering the temperature to 25°C, splicing defects of these introns recovered in wt cells, but the recovery with *whi5* and *atg20* introns was slower in *Δsde2*. The heat-sensitive splicing defects and the slower recovery of splicing in *Δsde2* seem to correlate with the presence of secondary structures between the BP and 3′ss. This defect could be attributed to the presumed opening of secondary structures resulting in increased effective distances between the BP and 3′ss. Excision of introns lacking secondary structures [e.g. *tho5* intron 1; introns in *vps55* encoding a vacuolar sorting protein (41); and introns in *hse1* encoding the ESCRT-0 complex subunit (42)] did not appear heat sensitive, suggesting optimal spliceosomal activity (Supplementary Figure S7B). Structured introns such as *cam1* intron 1, *ste4* intron 1 and *fmd1* intron 2 did not show temperature-sensitive splicing, suggesting that the heat shock regimes tested above might not have opened their structures. Also, structures in introns are likely to have specific regulatory elements and roles beyond RNA splicing.

**Figure 5.**
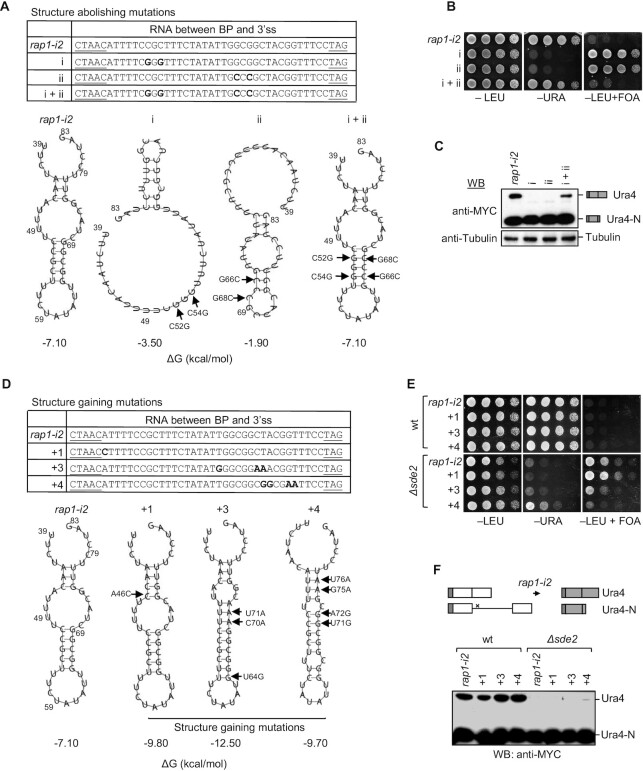
RNA structures may bring the 3′ss closer to the BP. (**A**) Predicted secondary structures of the RNA between the BP and 3′ss in *rap1*-*i2* variants. Arrows indicate nucleotides where mutations were made in the *rap1*-*i2* construct. The bold letters in the table indicate the mutations, and the underlined letters indicate the BP and 3′ss. (**B**) *S. pombe* growth on the indicated plates with *rap1-i2* reporter variants (incubation time: –LEU, –URA, 3 days; +FOA, 4 days). (**C**) Western blot analysis to check for splicing defects with different *rap1-i2* reporter variants. (**D–F**) The assays with the *atg20-i2* reporter variant are similar to (A–C). –LEU and –URA plates were scanned after 3 days, and +FOA plates after 4 days.

Specific Sde2 target introns, including 39 nt RNA between the BP and 3′ss of *rap1* intron 2, were also predicted to fold into weak secondary structures (Supplementary Figure S8). To test whether the secondary structures could affect pre-mRNA splicing, *rap1* intron 2 was mutated to weaken the structure. The effective distance between the BP and 3′ss was predicted to increase by the change. Weakening of the structure caused splicing defects even in wt *S. pombe*. Defects due to altered bases could be rescued by regaining an RNA fold through complementary mutations (Figure 5A–C). Changes were also introduced to stabilize the structure in *rap1* intron 2 to increase the abundance and lifetime of folded transcripts. (Figure 5D–F). RT–PCR and qRT-PCR analyses showed that excision of structurally stabilized *rap1-i2* improved in *the Δsde2* strain (Supplementary Figure S9A, B). These results suggested that Sde2 activities and potential RNA structures synergize splicing of introns with BP-distant 3′ss. To independently verify the above data in another target, mutations were introduced to abolish the predicted structure in *atg20* intron 2. Excision of this intron variant became Sde2 dependent. Furthermore, complementary mutations that regain an RNA fold in the variant intron also partially rescued the splicing defect in *Δsde2* (Supplementary Figure S9C–E).

### Sde2 functions with Cactin/Cay1 and Tls1

**Figure 6.**
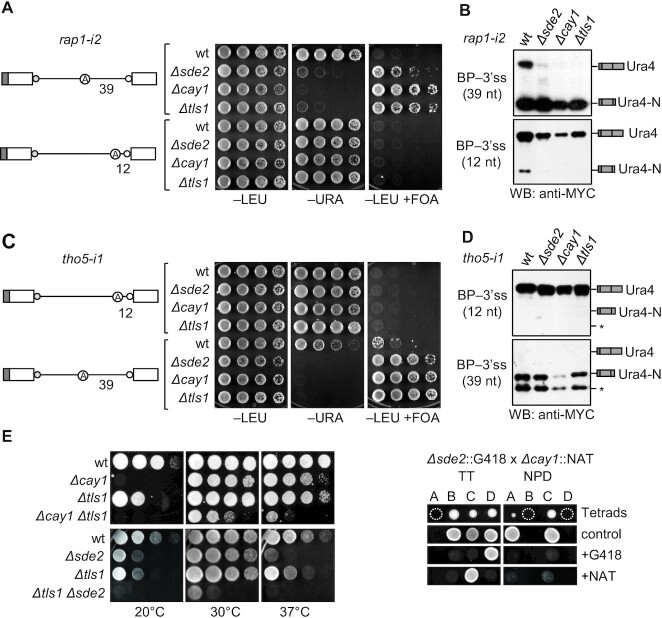
Sde2 functions with Cactin/Cay1 and Tls1. (**A** and **B**) Growth assay and immunoblot analysis showing introns with a longer distance between the BP and 3′ss also require Cay1 and Tls1, similar to Sde2. Reducing the distance in *rap1*-*i2* makes its splicing independent of these factors (incubation time: –LEU, –URA, 3 days; +FOA, 4 days). (**C** and **D**). The growth assay and immunoblot analysis show that increasing the distance in the *tho5*-*i1* reporter makes its splicing dependent on Cay1 and Tls1, as Sde2. * marks proteins arising from aberrant splicing or proteolytic cleavage (incubation time: –LEU, –URA, 3 days; +FOA, 4 days). (**E**) Genetic interaction among intron-specific splicing factors. The double mutants *Δsde2 Δtls1* and *Δtls1 Δcay1* grow more slowly in comparison with the respective single mutants at all temperatures. We crossed *Δsde2* and *Δcay1*, and the lack of *Δsde2 Δcay1* mutants [expected at (A) in TT and at (B) and (D) in NPD] indicates the synthetic lethality of the double mutant. Plates at 30°C and 37°C were scanned after 3 days, and the plate at 20°C was scanned after 5 days. TT, tetratype; NPD, non-parental ditype.

Besides Sde2, additional splicing factors might facilitate the usage of a BP-distant 3′ss. Thus, we searched for more factors needed for splicing the BP-distant 3′ss *rap1* intron 2. Splicing of *rap1-i1* and *rap1-i2* reporters was tested in a collection of 48 viable deletion mutants of putative splicing factors and 10 conditional mutants of splicing factors essential for viability (Supplementary Figure S10A, B). Assaying the activities of both reporters in the above mutants allowed us to exclude constitutive splicing factors, including the ones involved in splicing *rap1* intron 1 (mutants defective in constitutive splicing would not splice either reporter). Reporter-specific growth assays could not be used under restrictive conditions of mutants in essential factors (when their spicing defects become visible). Therefore, to identify *Δsde2*-like defects in these mutants, the splicing of Sde2 target introns, *rap1* intron 2 and *mcs2* intron 2, was assayed by RT–PCR (Supplementary Figure S10B) [Mcs2 is a TFIIH complex cyclin (43)]. Among the mutants studied, *Δcay1* and *Δtls1* strains also showed splicing defects specific for *rap1* intron 2 (Supplementary Figure S10A). The defects in *Δcay1* and *Δtls1* were also specific to introns with a BP-distant 3′ss. Shortening the distance to 12 nt made the splicing independent of all three factors (Figure 6A, B). In contrast, the *tho5-i1* reporter with the BP and 3′ss 12 nt apart did not require these factors but became dependent on all three when the BP was shifted 39 nt away from the 3′ss (Figure 6C, D). These results suggested that Sde2, Cay1 and Tls1 play a common role in facilitating the usage of a BP-distant 3′ss.

We assessed genetic interactions between *sde2*, *cay1* and *tls1* by combining their deletions into double mutants. These factors showed negative genetic interactions; co-deletion of any two genes in the cell was synthetically sick or probably lethal (Figure 6E). These phenotypes suggested that the three factors acted separately. Overexpression of one protein did not complement defects from the lack of another, although overexpression of Tls1 enhanced growth in the *Δsde2* strain (Supplementary Figure S11A). Thus, the splicing activities of these proteins were not redundant. Although Sde2 helps recruit Cay1 to the spliceosome (22), yeast two-hybrid assays did not detect interactions between Sde2, Cay1 and Tls1 (Supplementary Figure S11B), suggesting that these proteins may not bind to one another directly. The genetic relationships between these factors were further verified by monitoring the splicing of the reporters in the respective double mutants. Like single mutants, the viable double mutants could efficiently splice the *rap1-i1* reporter, but not *rap1-i2* (Supplementary Figure S11C). Concerning the BP–3′ss distance threshold required for splicing, *Δcay1 Δtls1* and *Δsde2 Δtls1* double mutants showed splicing defects similar to their single mutants (Supplementary Figure S11D).

### Sde2, Cay1 and Tls1 control the expression of RNA interference (RNAi) and heterochromatin factors via splicing

**Figure 7.**
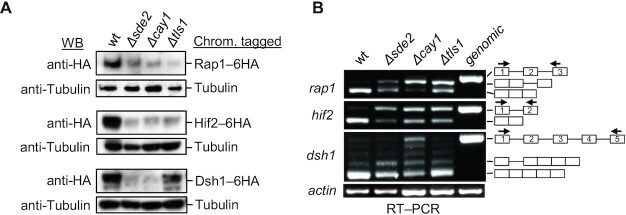
Sde2, Cay1 and Tls1 control the expression of heterochromatin factors through intron-specific pre-mRNA splicing. (**A**) Rap1, Hif2 and Dsh1 proteins are lower in deletion mutants of intron-specific splicing factors. Immunoblotting was performed for the chromosomally C-terminal 6HA-tagged strains using an anti-HA antibody. (**B**) Semi-quantitative RT–PCR was performed to assay the splicing of heterochromatin factors in the indicated strains. Arrows in the schematic indicate where the primer binds.

Spliceosomes use Sde2, Cay1 and Tls1 to splice pre-mRNAs containing introns with BP-distant 3′ss, whereas introns with BP-near 3′ss in the same pre-mRNA do not require these factors. These proteins have also been involved in heterochromatin silencing (25,44,45). Tls1 was reported to regulate the expression of genes of the shelterin complex via pre-mRNA splicing. In contrast, splicing factors have also been shown to act in heterochromatin silencing independently of their splicing functions (46–48). These factors could regulate heterochromatin formation via splicing across BP-distant 3′ss introns. Thus, we monitored protein levels of selected Sde2 targets functioning at the chromatin level. A 6HA epitope tag was inserted at the C-termini of the genes at their chromosomal loci in *S. pombe*. The genes *rap1*, *bqt3*, *dsh1*, *hif2*, *pst2* and *rxt2* were tagged in the wt, *Δsde2, Δcay1* and *Δtls1* strains (Supplementary Figure S12A). Anti-HA western blots showed diminished protein levels of key heterochromatin factors [Rap1, RNAi factor Dsh1 (49); Set3 histone deacetylase complex protein Hif2 (50); and histone deacetylase complex subunits Rxt2 (51) and Pst2 (52); and the telomere bouquet-forming protein Bqt3 (53)] in all three mutants (Figure 7A; Supplementary Figure S12B). RT–PCR showed retention of specific introns in the above heterochromatin and RNAi factors in *Δsde2, Δcay1* and *Δtls1* strains (Figure 7B).

Protein levels of Mcs2, Hif2, Psf3, Rxt2 and Rap1 were also monitored by expressing N-terminal 3MYC epitope-tagged genomic constructs in wt and *Δsde2* strains. These proteins were lower in the *Δsde2* strain, further confirming the role of Sde2 in gene expression. As expected, the full-length Rap1 protein diminished in *Δsde2*, but an alternative Rap1 protein of ∼19 kDa (Rap1-N) was detected in this strain. Similarly, the full-length Rxt2 protein decreased in *Δsde2*, but an alternative form of ∼13 kDa accumulated (Supplementary Figure S13A). These results indicated that controlled BP-distant 3′ss usage could promote alternative splicing through intron retention.

Since an alternative Rap1-N protein accumulated in *Δsde2, Δcay1* and *Δtls1* mutants, we used Rap1-N as a measure to find strains with similar phenotypes in the mutant collection discussed earlier (Supplementary Figure S10A). Rap1-N was also detected in several other splicing mutants, although its accumulation was higher in *Δsde2, Δcay1* and *Δtls1* (Supplementary Figure S13B, C). RT–PCR assays in selected strains also detected transcripts containing *rap1* intron 2 (Supplementary Figure S13D). Rap1-N is expected to be translated from the intron 2-retained mRNA variant with a translational stop codon in the intron (Supplementary Figure S13B). We confirmed Rap1-N’s origin by mutating the stop codon. The change abolished the protein (Supplementary Figure S14A). Therefore, the alternative protein would have the N-terminal 157 amino acids of Rap1 containing the BRCT domain and a part of the Myb domain. A function has not been assigned to this segment of Rap1 protein (54).

The loss of Rap1 in *S. pombe* is reported to produce defective silencing of telomeric reporters and aberrant expression of telomeric transcripts (44). However, ectopic expression of Rap1-N in a telomeric reporter strain did not reveal silencing defects (Supplementary Figure S14B). Therefore, the physiological significance of Rap1-N remains unknown. We also tested whether Rap1-N would accumulate under certain conditions by expressing the 3MYC epitope-tagged *rap1* genomic clone in wt *S. pombe*. Protein accumulated at high temperature and after treatments with hydroxyurea (HU), cadmium and sorbitol (Supplementary Figure S14C). This accumulation could result from splicing defects arising from the sensitivity of structured RNA between the BP and 3′ss of *rap1* intron 2. Thus, intronic diversity and dedicated splicing regulators of the kind discussed in this study allow the cell to control gene expression and promote alternative splicing to create protein variants that help the cell under specific conditions.

Thus, we propose a model based on our findings and structural studies (6,43) explaining the spliceosomal processing of BP-distant 3′ss. The two signals could be brought into proximity by two potentially related mechanisms. With the help of Sde2, Cay1 and Tls1, and RNA secondary structures, the spliceosome could position extra RNA between the BP and 3′ss into the complex (Supplementary Figure S15; also see the Discussion). The model has also proposed an alternative possibility that these factors function as post-mRNA release factors.

## DISCUSSION

### The regulators of BP-distant 3′ss usage in *S. pombe*

This study shows that Sde2, Cay1 (Cactin in humans) and Tls1 (C9ORF78/hepatocellular carcinoma-associated antigen 59 in humans) form a specific group of spliceosomal regulators that facilitate the use of BP-distant 3′ss. These proteins were previously reported to function in pre-mRNA splicing (22,45,55–58), but their specificity for target pre-mRNAs or introns was unknown. Their intron-specific splicing function was revealed in two independent approaches in this study. The first approach monitored the activities of *S. pombe ura4* splicing reporters harbouring introns with a BP-near and BP-distant 3′ss in a collection of *S. pombe* mutants. A similar reporter with *nda3* introns was used to study exon skipping in *S. pombe* (26). The reporters were assayed by three techniques: growth, RT–PCR and western blot, which collectively made the outcome more sensitive and specific. The second approach detected an alternatively spliced form of the telomeric factor Rap1 resulting from retention of its second intron due to poor usage of a BP-distant 3′ss.

The splicing with a BP-distant 3′ss became independent of these factors once the spacing between the BP and 3′ss was reduced. Intrinsic choice of the spliceosome for a BP-near 3′ss made by essential RNA-binding proteins (59–65) dominated over activities of these regulators; a BP-near 3′ss was preferred even in the presence of Sde2, Cay1 and Tls1. Despite obvious growth defects, none of these factors was essential for the viability of *S. pombe* under standard growth conditions, and none was needed for constitutive splicing. Therefore, the activities of these regulators improve splicing efficiency, specifically of pre-mRNAs containing introns with a BP-distant 3′ss. Genetic interactions among Sde2, Cay1 and Tls1 confirmed the specificity of these factors. Like single deletion mutants, splicing defects in double mutants were also specific to such introns. However, these molecules do not appear to have fully overlapping functions since the growth phenotypes of individual deletion mutants were distinct. Also, they could function beyond pre-mRNA splicing. Sde2, for example, is reported to regulate the replication stress response (21,31,66,67) and ribosome biogenesis in humans (20) and anthocyanin biosynthesis in *Arabidopsis* (68).

### An added layer of controls for BP-distant 3′ss usage

Sde2, Cay1 and Tls1 appear to act beyond the known mechanisms of BP-distant 3′ss usage in *S. cerevisiae*. Although conserved from *S. pombe* to humans, these molecules are absent in *S. cerevisiae*. The BP and 3′ss are separated by an average spacing of 36 nt in *S. cerevisiae* and 29.3  ±  11.9 nt in humans. (9,69–71). The optimal distance between the BP and 3′ss in humans is 19–23 nt (65). The conserved and essential splicing factors Slu7 and Prp18 are known to promote the usage of a BP-distant 3′ss, not only in *S. cerevisiae* (72–75) but also in *S. pombe* (60). Distant 3′ss in *S. cerevisiae* are also selected by reducing the effective distance between the BP and 3′ss through RNA folding (76–78). However, despite highly optimized introns, uniformity in splicing and the presence of the above mechanisms of 3′ss selection, *S. cerevisiae* spliceosomes used all available 3′ss within a distance of 10–45 nt (46) [though BP-proximal 3′ss were preferred (79)]. This mode of BP-distant 3′ss selection could result in error-prone splicing. The activities of Sde2, Cay1 and Tls1 were possibly retained in intron-rich organisms for added layers of control to avoid errors caused by competing branch points or 3′ss. Therefore, intron-rich eukaryotes use (at least) two mechanisms to excise introns with a BP-distant 3′ss through RNA structures and specific regulators of the spliceosome, and the two mechanisms are likely to be complementary.

### Potential mechanism of Sde2, Tls1 and Cay1

The activities of Sde2, Cay1 and Tls1 that make spliceosomes competent for BP-distant 3′ss need further investigation. We propose that these factors could guide the incoming 3′ss towards the spliceosome's catalytic centre. Loss of these factors led to the retention of selected introns. The *sde2* deletion also resulted in mild splicing defects for introns with a non-canonical BP and 3′ss, suggesting that Sde2 may act with U2 snRNP and U2AF that recognize the BP and 3′ss. This possibility is also suggested by the homology between mammalian Sde2 and the U2 snRNP assembly factor SF3A3 (80–82). However, as discussed below, these regulators are more likely to act once splicing signals have been recognized. *Schizosaccharomyces pombe* Sde2-C facilitates the recruitment of Cay1/Cactin to the spliceosome (22), a result supported by the cryo-electron microscopy structure of the human post-catalytic spliceosome (6). The structure shows that Sde2-C and Cactin function as exon ligation factors by stabilizing the branch helix in a suitable conformation for the ligation.

Sde2, Cay1 and Tls1 may facilitate the positioning of the RNA intervening the BP and 3′ss. The RNA between the BP and 3′ss must loop during exon ligation to juxtapose the two exons in the catalytic centre. This RNA structure in the spliceosome is yet to be seen (5,6,83), but its flexible or longer trajectories could hinder the spliceosome by slowing down the docking of the incoming 3′ss in the catalytic centre. Indeed, *tho5* intron 1 variants with longer gaps between the BP and 3′ss were poorly excised even in wt cells. A positively charged amino acid patch in human Cactin was proposed to follow a path predicted for the RNA between the BP and 3′ss (6). The role of this surface might become more critical with longer RNAs between the two sites.

Furthermore, these factors could also act by modulating the RNA structures between the BP and 3′ss. Tls1 has been reported to bind Brr2 (a U5-snRNP-specific RNA helicase that unwinds U4/U6 snRNA duplexes during spliceosomal activation) in *S. pombe* and humans (45,84). Tls1 has been shown to regulate Brr2’s activity in humans (84). The helicase has been proposed to play an additional role in the second step of splicing via substrate repositioning. A Brr2 mutant showed second step splicing defects for BP-distant 3′ss introns and introns with potential secondary structures between the BP and 3′ss (85). Thus, the Tls1–Brr2 interaction and the roles of the two proteins in the BP-distant splicing suggest that Tls1 could regulate Brr2 activity on RNA structures between the BP and 3′ss. Increasing the effective distance between the BP and 3′ss by weakening the RNA structure through temperature stress aggravated the splicing defect of *rap1* intron 2, whereas decreasing the effective gap by strengthening the structure alleviated the defect.

Notwithstanding the above evidence, definitive proof for Sde2, Cay1 and Tls1 function can come from structures where these factors and the RNA intervening the BP and 3′ss are resolved. However, we cannot rule out the possibility that these factors play a role in spliceosomal disassembly post-catalysis. Similar activity has been ascribed to the RNA helicase Prp22 in *S. cerevisiae*; the enzyme promotes the disassembly of the spliceosome in *S. cerevisiae*. Extracts lacking Prp22 also showed defects in the splicing of BP-distant 3′ss introns (86,87).

### The physiological relevance of splicing through BP-distant 3′ss

We report that Sde2, Cay1 and Tls1 control gene expression and alternative splicing of selected chromatin and RNAi factors in *S. pombe*. We and others have shown that their mutants are defective in heterochromatin silencing and genomic instability (22,37,42,45,47,78). RNAi defects in the *sde2* mutant have also been reported (47). These phenotypes could result from defects in the expression of chromatin factors, e.g. the shelterin complex subunit Rap1, the histone deacetylases Hif2 and Rxt2, or the RNAi machinery assembly factor, Dsh1. The Sde2 mutant also accumulated alternative forms of Rap1 and Rxt2. The function of the alternative Rap1 and Rxt2 has not yet been elucidated. Although their aberrant origin cannot be ruled out, these proteins might function only under specific conditions. Indeed, the alternative Rap1 accumulated in wt cells under chemical and temperature stress. Sde2, Cay1 and Tls1 could also make gene expression and alternative splicing conditional or tissue specific in multicellular eukaryotes rich in diverse introns. Indeed, mammalian introns with BP-distant 3′ss have a higher tendency to undergo alternative splicing (88,89). Further regulation of splicing by these factors is plausible, considering a stringent control over Sde2 protein, involving its activation by DUB and degradation by the proteasome (21,22,31).

Heat-sensitive splicing of the structured *S. cerevisiae APE2* intron is reported to work like a thermosensor (78). Notably, splicing defects upon heat shock could arise from multiple causes; among them may be the potential opening of RNA structures between the BP and 3′ss. Splicing of selected pre-mRNAs with possible structures between the BP and 3′ss appeared sensitive to elevated temperatures, suggesting that BP-distant 3′ss may play regulatory roles in gene expression. Similar sensitivities could be expected with cellular metabolites that modulate RNA structures. Mutants of several splicing factors, including *Δsde2* and *Δcay1*, show sensitivities to low and high temperatures, metabolites or chemicals such as formamide, possibly for this reason (25,44,90). It would be interesting to check if some of the structured introns studied here could work as sensors for ions, chemicals or heat. Also, chemical probing should be done to confirm the presumed folding in the introns. Another interesting example of gene regulation by intronic RNA is the intron in *S. pombe* telomerase RNA *ter1* (91). This RNA controls its own maturation. The sequence between the BP and 3′ss impedes exon ligation but activates a discard pathway essential for *ter1* maturation. Another class of introns with RNA structures prevalent in zebrafish genes has been reported. Splicing of these structured pre-mRNAs was independent of the essential U2AF2 protein (92).

In conclusion, Sde2, Cactin/Cay1 and Tls1 bring more advanced controls over pre-mRNA splicing involving introns with a BP-distant 3′ss. Activities of these regulators appear in sync with the RNA structures to bring the BP and 3′ss closer to each other in the spliceosome. These processes become more relevant to intron-rich organisms because they add an extra layer of specificity and control over gene expression. Such regulations would become critical when the cell is faced with suboptimal conditions. Furthermore, given the vast diversity of introns available in eukaryotic genomes, these controls would also allow spliceosomes to receive more messages from the existing gene pools through alternative splicing.

## DATA AVAILABILITY

All data are available in the main text or the supplementary materials. The microarray data analysed in this study is available in Gene Expression Omnibus (GEO) database under the accession number GSE97097.

## Supplementary Material

gkac769_Supplemental_File

## Notes

Present address: Karan Choudhary, The George S. Wise Faculty of Life Sciences, Department of Molecular Microbiology and Biotechnology, Tel Aviv University, Ramat Aviv, 69978, Israel.

Present address: Praver Gupta, TIFR Centre for Interdisciplinary Sciences (TCIS), Tata Institute of Fundamental Research Hyderabad, 500107, Hyderabad, India.

Present address: Poonam Thakran, Laboratory of Ribonucleoprotein Biochemistry, National Heart, Lung and Blood Institute, National Institutes of Health, Bethesda, MD 20814, USA.

Present address: Arashdeep Singh, Cancer Data Science Lab, National Cancer Institute, National Institute of Health (NIH), Bethesda, MD 20892, USA.

Present address: Monika Sharma, Biological Systems Engineering, Plaksha University, Sector 101 Alpha, IT City, SAS Nagar, Punjab 140306, India.
