# Supplementary material for: Splicing of branchpoint-distant exons is promoted by Cactin, Tls1 and the ubiquitin-fold-activated Sde2
Source: Nucleic Acids Res. 2022 Sep 12;50(17):10000–14. doi: 10.1093/nar/gkac769 (PMC9508853; doi:10.1093/nar/gkac769)
Supplement: gkac769_Supplemental_File [file gkac769_supplemental_file.pdf]

SUPPLEMENTARY INFORMATION

Splicing of branchpoint-distant exons is promoted by Cactin, Tls1, and the ubiquitin-fold-activated Sde2

Anupa T. Anil[1¶], Karan Choudhary[1,3¶], Rakesh Pandian[1¶], Praver Gupta[1,4], Poonam Thakran[1,5], Arashdeep Singh[1,6], Monika Sharma[2,7], and Shravan Kumar Mishra[1*]

[1]Department of Biological Sciences and [2]Department of Chemical Sciences, Indian Institute of Science Education and Research (IISER) Mohali, Sector 81, 140306 Punjab, India

[¶]Co-first authors

*To whom correspondence should be addressed. E-mail: skmishra@iisermohali.ac.in, mishra.iiserm@gmail.com

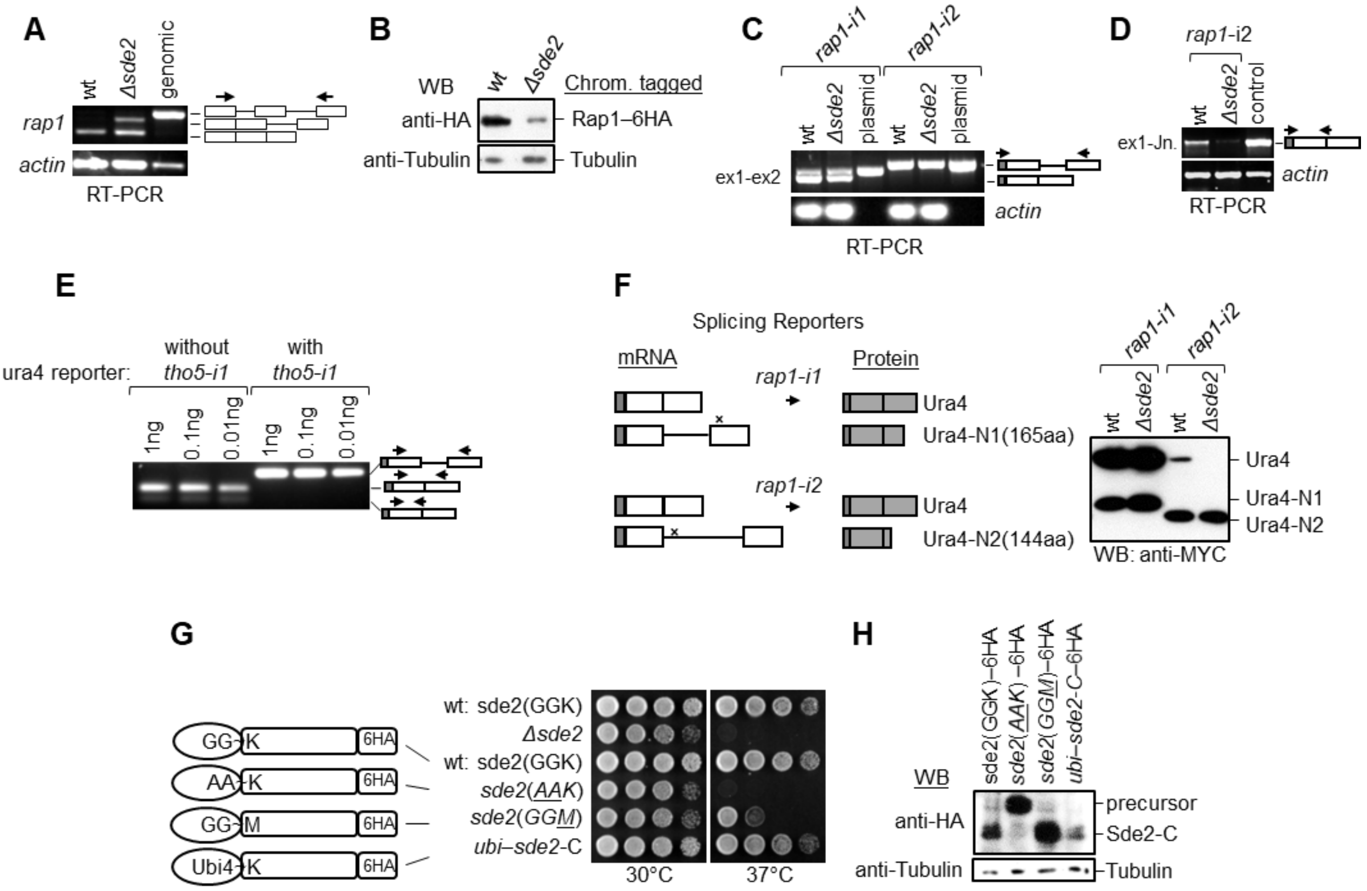

Figure S1. Intronic features define Sde2's role in pre-mRNA splicing.

(A) Semiquantitative RT-PCR shows retention of *rap1-i2* in *Δsde2* strain. Arrows, blocks, and lines indicate primers, exons, and introns. Genomic DNA from *S. pombe* was used as a control to size intron-containing transcript.

(B) Immunoblot analysis to detect Rap1 tagged with chromosomal epitope 6HA using anti-HA antibodies. Rap1 protein level is lower in *Δsde2* strain compared to wt.

(C) Semiquantitative RT-PCR shows the intron-retained band for *rap1* reporters. The signal corresponding to *ura4* cDNA was not detectable for the *rap1-i2* reporter in this assay. *rap1-i1* and *rap1-i2* reporter plasmids were used as a control to size intron-containing transcripts. ex1-ex2 indicates PCR performed using the exon1 forward primer and exon2 reverse primer. Arrows in the schematic indicate the primers' binding site.

(D) Semiquantitative RT-PCR shows decreased *ura4* cDNA from the *rap1-i2* reporter in the *Δsde2* strain. ex1-Jn. signal indicates PCR performed using the forward primer specific to the *myc* epitope and the reverse primer specific to the *ura4* exon1-exon2 junction. This amplification is specific for spliced transcripts.

(E) Multiplex-PCR to show the specificity of the junction primer. Indicated amounts of intron-less and intron-containing reporter plasmids were amplified with a forward primer annealing to exon1 and reverse primers annealing to exon2 and exon1-exon2 junction.

(F) Schematic represents protein formed when the intron is spliced or unspliced in the *rap1-i1* and *rap1-i2* reporters. *Δsde2* strain lacks Ura4 protein originating from the *rap1-i2* reporter. Immunoblot analysis was performed using anti-MYC antibodies to detect the Ura4 protein. Numerical in parenthesis indicates the size of the protein, and 'x' denotes the stop codon that arises due to retention of the intron that causes a change in the translation reading frame. Ura4 -N1 and -N2 indicate proteins from mRNAs with exon1 until the stop codon.

(G,H) Growth assay and Immunoblot analysis of Sde2 variants used in Figures 1E and 1F. Plates were scanned after three days of incubation. Mutation sites are underlined. The *AAK* and *GGM* variants of *sde2* could not complement the temperature sensitivity of *Δsde2,* whereas *ubi–sde2-C* that generates activated sde2-C could complement.

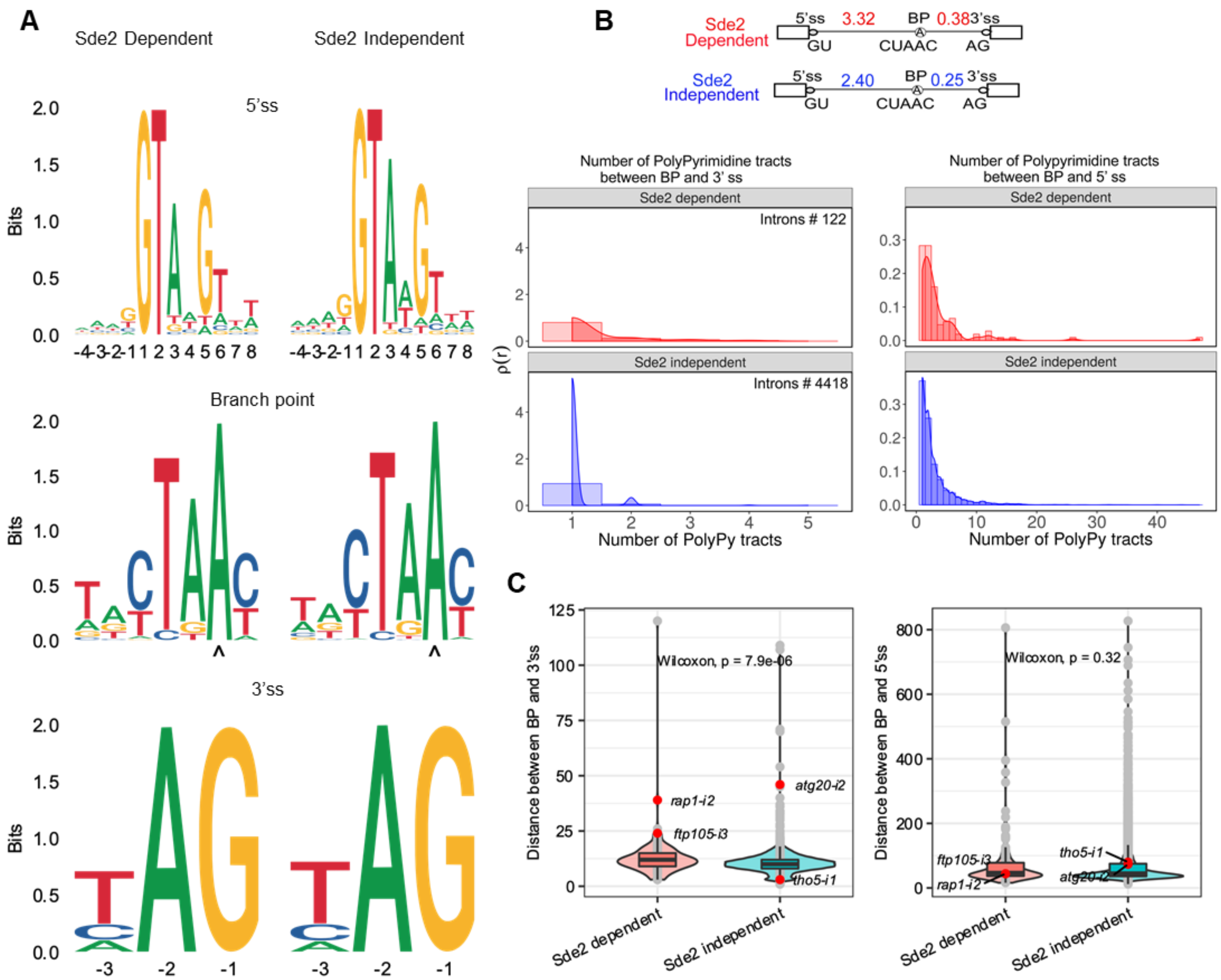

Figure S2. Bioinformatic analysis between Sde2 dependent and independent introns.

(A) Sequence logos for 5'ss, branch point, and 3'ss for Sde2 dependent and independent introns using the ggseqlogo package in R. Violin plot showing the distribution of the distance between 5'ss and BP and BP and 3'ss for Sde2 dependent and independent introns.

(B) Occurrences of the polypyrimidine tract in Sde2-dependent and Sde2-independent introns. The number in the schematic shows the average number of polypyrimidine tracts (defined as at least six consecutive non-adenine nucleotides containing no fewer than three uridines present per intron) between BP and 3'ss and BP and 5'ss.

(C) Violin plot showing the distribution of distances (number of nucleotides) between the branch point (BP) adenosine and the 5' and 3'-splice sites (ss) of the Sde2-dependent and -independent introns. The outlier in the violin plot is *sac3* intron-1 (a Sde2-dependent intron) which has a distance of 120bp between BP and 3'ss.

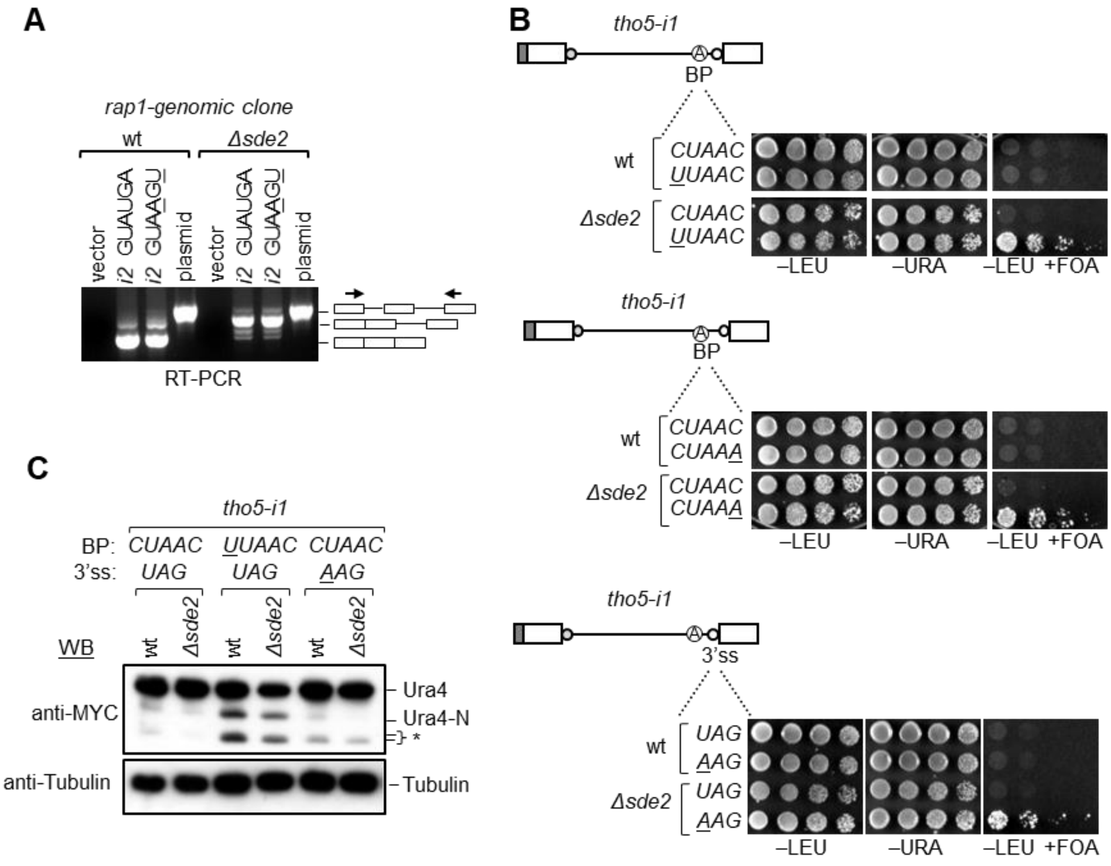

Figure S3. Usage of suboptimal splice sites in *Δsde2.*

(A) Semiquantitative RT-PCR showing the splicing of *rap1* on making the 5'ss optimal in the wt and Δsde2 strain. Underline indicates the mutation incorporated to make the 5'ss optimal. Arrows indicate primers used for the assay.

(B) Growth of indicated strains with BP and 3'ss mutants of the *tho5-i1* reporter (incubation time: -LEU, -URA - 4 days; +FOA - 5 days).

(C) Immunoblot with different variants of BP and 3'ss mutant in the *tho5-i1* reporter. Underline indicates mutations.

A

5'ss-BP	*rap1-i2*
44	GTATGATCTTGCTTACCATTAATTGTTTTTTTATTTTTTT--------------------------TT**CTAA**CATTTTCCGCTTTCTATATTGGCGGCTACGGTTTCCTAG
70	GTATGATCTTGCTTACCATTAATTGTTTTTTTATTTTTTTAGATTTCAAAAGTCTTTGTTCATTTCTTT**CTAA**CATTTTCCGCTTTCTATATTGGCGGCTACGGTTTCCTAG

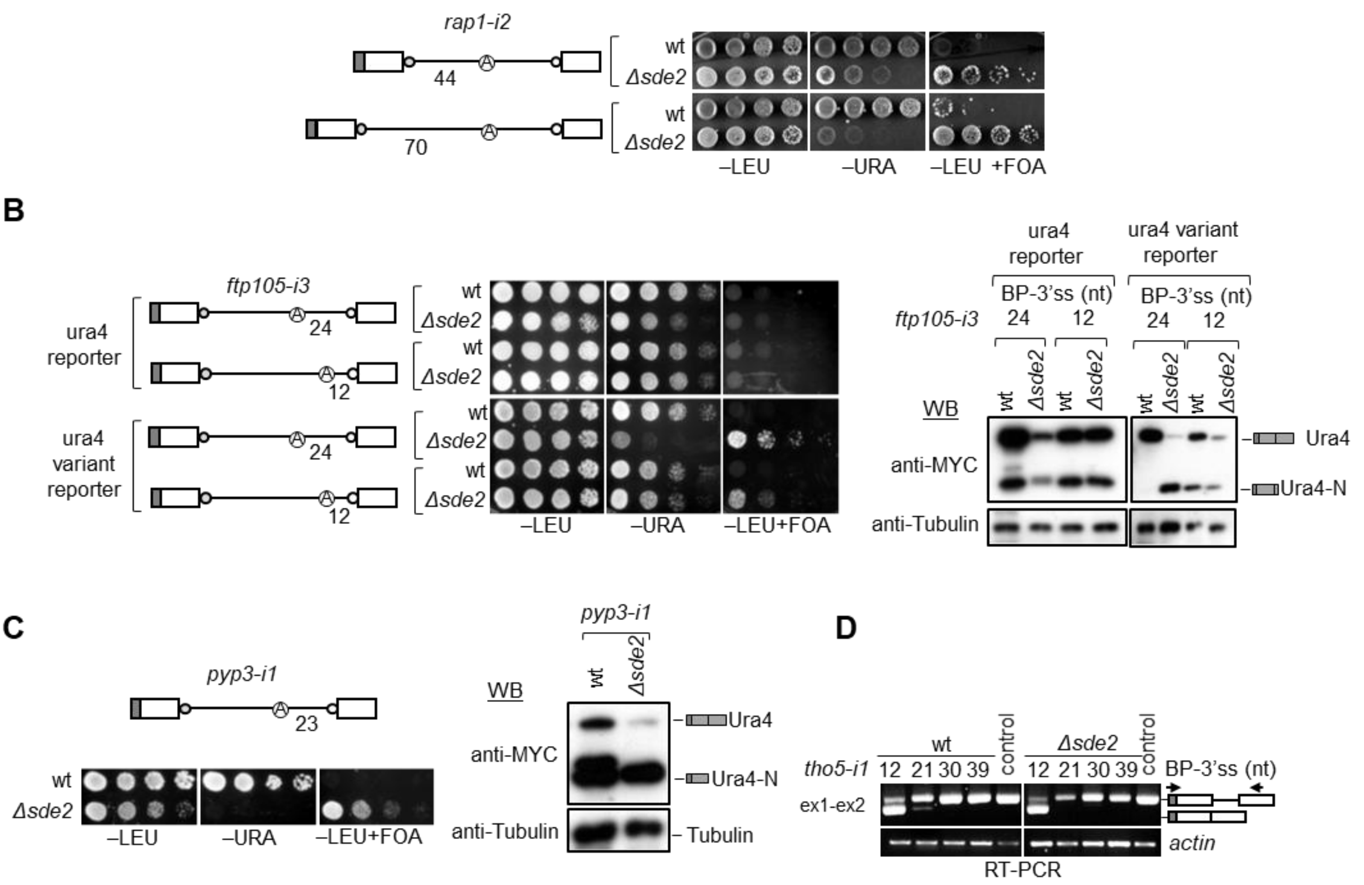

Figure S4. Sde2 targets are introns with longer spacing between BP and 3'ss

(A) Growth of indicated strains with constructs that have a different distance between 5'ss and BP. As shown in the schematic, 26 nt were introduced between 5'ss and BP. The underline represents where the nucleotides were inserted (incubation time: -LEU, -URA - 4 days; +FOA - 5 days).

(B) Growth and immunoblot of the indicated strains with the *ftp105-i3* reporter with different distances between BP and 3'ss. A ura4 variant *ftp105-i3* reporter was used to make the assay more sensitive (deletion of T nucleotide at the 786th position in *ura4* ORF led to a frameshift and addition of 40aa to the full-length *ura4* making it a hypomorphic mutant) (incubation time: -LEU, -URA - 3 days; +FOA - 4 days).

(C) Growth and immunoblot of the indicated strains with the *pyp3-i1* reporter (incubation time: -LEU, -URA - 4 days; +FOA - 4 days).

(D) Ura4 transcripts from different BP-3'ss distance reporters in wt and *Δsde2* were analysed using semiquantitative RT-PCR. ex1-ex2 indicates PCR performed using exon1 forward primer and exon2 reverse primer. The Intron-containing plasmid was used as a control to size the intron-retained band.

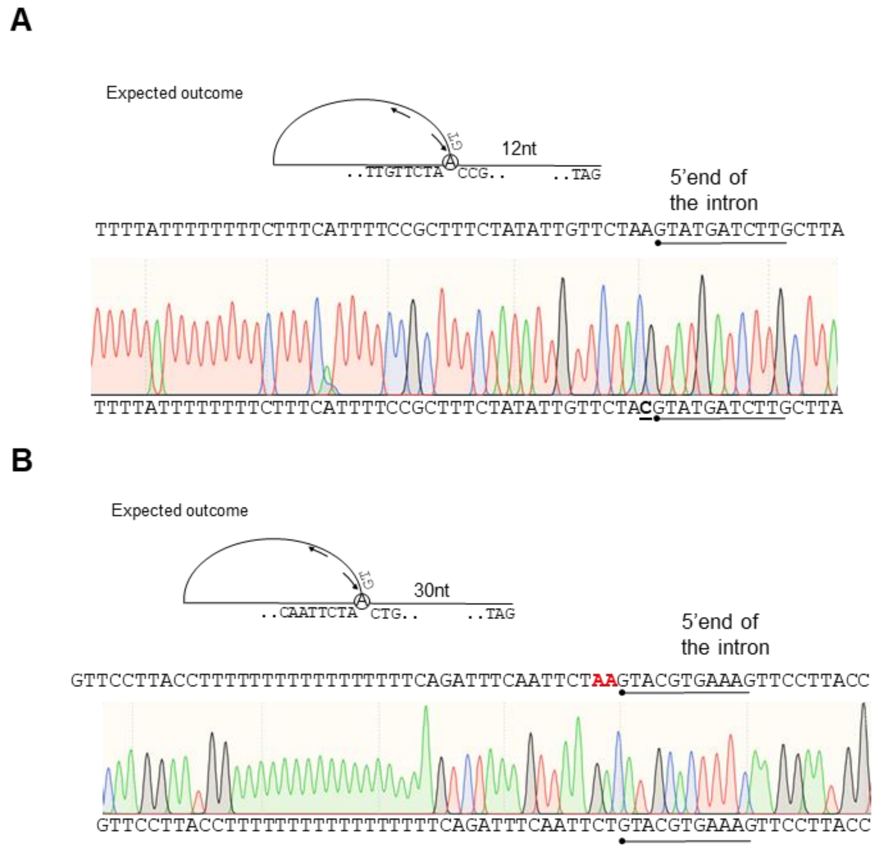

Figure S5. Usage of new BP in the reporter variant.

(A) Lariat-PCR sequencing to identify the BP used by the spliceosome in *tho5-i1* with a 30nt distance between BP and 3'ss, respectively. The primer in the schematic indicates the primer used for cDNA synthesis and for performing lariat RT-PCR. The underlined nucleotide shows sequenced lariat different from the expected outcome. This difference could have arisen because of the error-prone nature of reverse transcriptase in the presence of 3mM MgCl2 and 3mM MnCl2 (1).

(B) Lariat-PCR sequencing to identify the BP used by the spliceosome in *rap1-i2*. The nucleotide underlined indicate gives a different sequence from the expected sequence. The arrows in the schematic show the primer used for cDNA synthesis and for performing lariat RT-PCR. The nucleotides shown in red in the expected outcome were missing in the sequenced lariat.

Gene ID Intron number	BP-3’ss (nt)	$\log_2$ *Δsde2*/wt at 30°C	$\log_2$ *Δsde2*/wt at 37°C	Structure predicted by RNAfold web server	ΔG (Kcal/mol)
SPCC1884.01 Intron-1	27	-0.172	-0.074	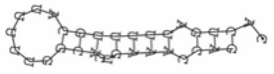	-3.70
Omh5, alpha-1,2-mannosyltransferase, SPBC32H8.08c Intron-1	88	-0.171	nd	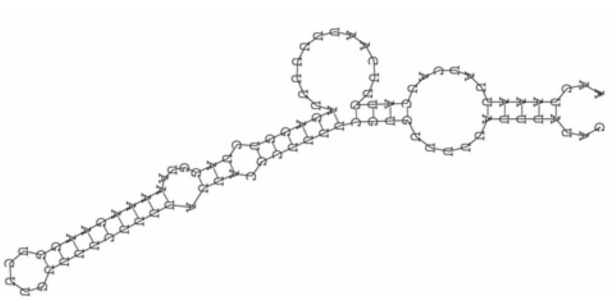	-8.10
SPCC1884.01 Intron-2	54	-0.08	-0.075	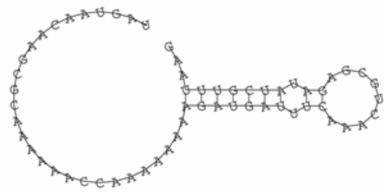	-4.70
Hop1, linear element protein, SPBC1718.02 Intron-3	25	-0.0629	nd	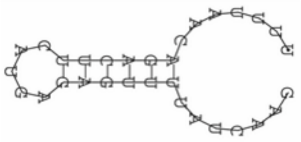	-2.10
Whi5, cell cycle transcriptional repressor, SPBC800.02 Intron-1	47	-0.046	-0.153	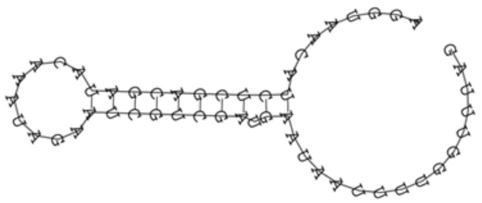	-10.40
Ulp2, SUMO peptidase, SPAC17A5.07c Intron-3	34	-0.022	0.137	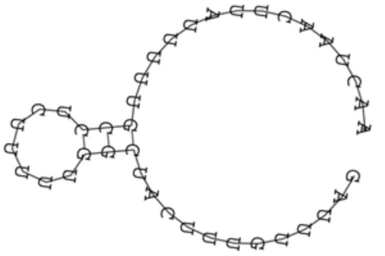	-4.00
NAD adenylyltransferase, SPAC694.03 Intron-1	21	0.024	nd	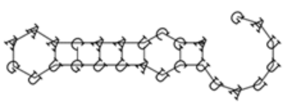	-1.90
Bet3, TRAPP complex subunit, SPAC644.18c Intron-1	24	0.028	-0.048	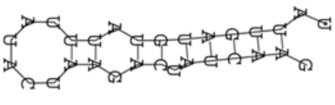	-1.80

Figure S6. Sde2-independent introns with BP-distant 3’ss and predicted structures of intervening RNA. (nd, not detected)

Predicted secondary structure of RNA between BP and 3’ss of Sde2 independent introns in the increasing order of splicing defect in *Δsde2* strain ($\log_2$ intron retention ratio *Δsde2*/wt at 30°C). The secondary structure was predicted using an RNAfold web server (2). ΔG refers to the Gibbs free energy change in kcal/mol.

Figure S6. (cont.)

Gene ID Intron number	BP-3'ss (nt)	log_2 *Δsde2*/wt at 30°C	log_2 *Δsde2*/ wt at 37°C	Structure predicted by RNAfold web server	ΔG (Kcal/ mol)
Ste4, adaptor protein, SPAC1565.04c Intron-1	102	0.023	0.016	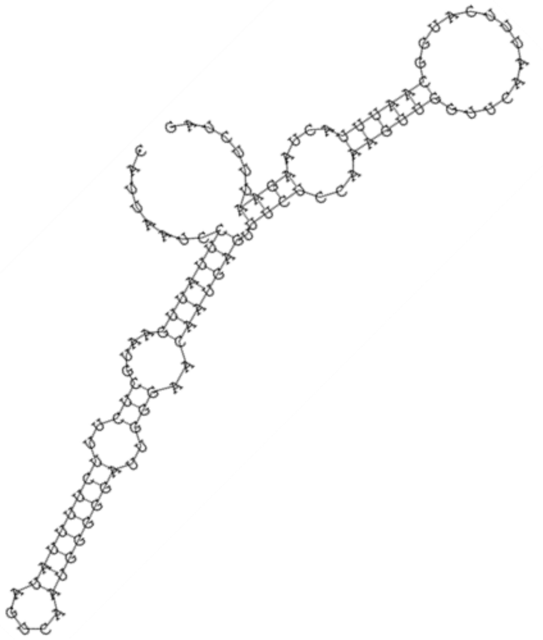	-16.30
Cox8, cytochrome c oxidase subunit VIII, SPAC24C9.16c Intron-1	24	0.035	0.002	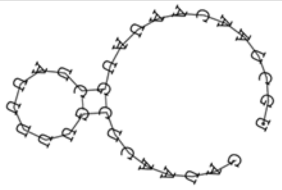	-0.70
Pir2, NURS complex subunit, SPBC725.08 Intron-1	28	0.049	0.303	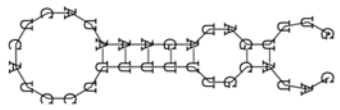	-0.90
Fmd1, glutathione-dependent formaldehyde dehydrogenase, SPBC1539.07c Intron-2	46	0.063	-0.045	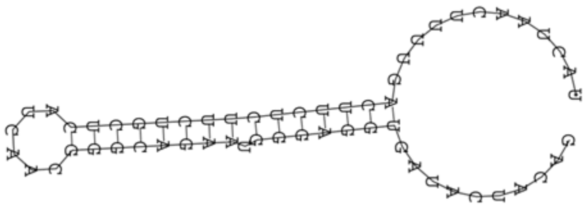	-20.50
Cam1, calmodulin, SPAC3A12.14 Intron-1	70	0.079	0.131	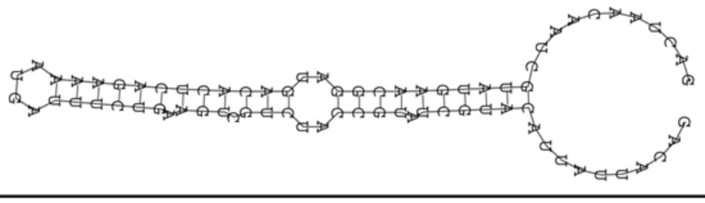	-22.70
Atg20, autophagy associated sorting nexin, SPCC16A11.08 Intron-2	46	0.086	0.201	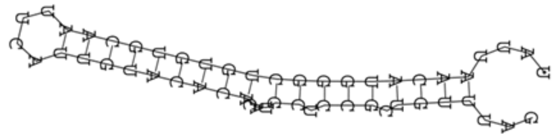	-16.30
Lea1, U2 snRNP protein, SPBC1861.08c Intron-1	21	0.091	0.05	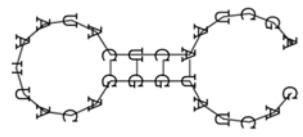	-1.40

Figure S6. (cont.)

Gene ID<br>Intron number	BP-3'ss (nt)	$\log_2$ *Δsde2*/wt at 30°C	$\log_2$ *Δsde2*/wt at 37°C	Structure predicted by RNAfold web server	ΔG (Kcal/mol)
Yop1, ER membrane, SPCC830.08c Intron-2	20	0.112	0.052	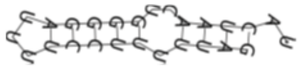	-4.20
Mug65, dysferlin-like membrane trafficking, SPAC1296.04 Intron-2	45	0.170	-0.10	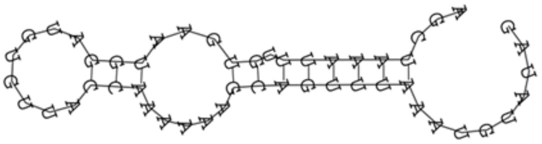	-3.50
Vma13, V-type ATPase V1 subunit H, SPAC7D4.10 Intron-5	107	0.201	0.234	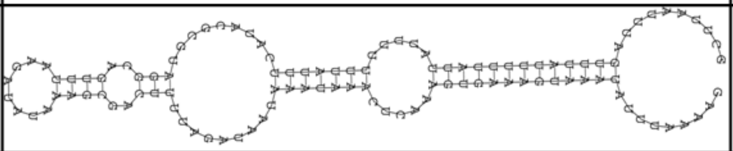	-13.40
Acyl-coenzyme A thioesterase SPAPB2B4.06 Intron-1	20	nd	0.232	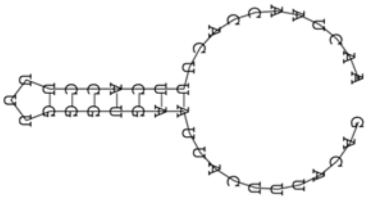	-0.70
Thg1, tRNAHis guanylyltransferase, SPCC63.07 Intron-2	33	0.255	0.017	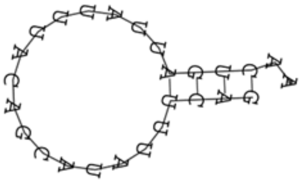	-7.40
Hst2, sirtuin family histone deacetylase, SPCC132.02 Intron-1	20	0.257	0.317	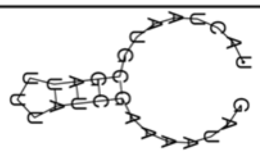	-1.30
Pof9, F-box, SPBC3H7.06c Intron-1	22	0.272	0.516	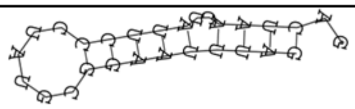	-4.40
Fcp1, CTD phosphatase, SPAC19B12.05c Intron-1	24	0.284	0.207	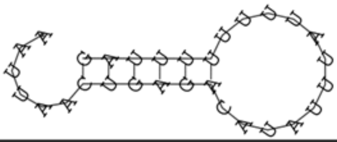	-0.30
Mbx1, MADS-box transcription factor, SPBC19G7.06 Intron-3	21	0.410	0.205	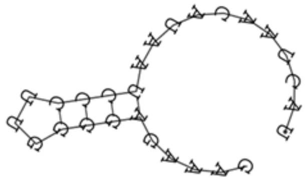	-3.90
Csn1, COP9/sigalosome subunit, SPBC215.03c Intron-1	40	nd	nd	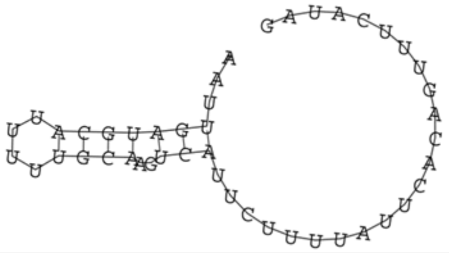	-4.20

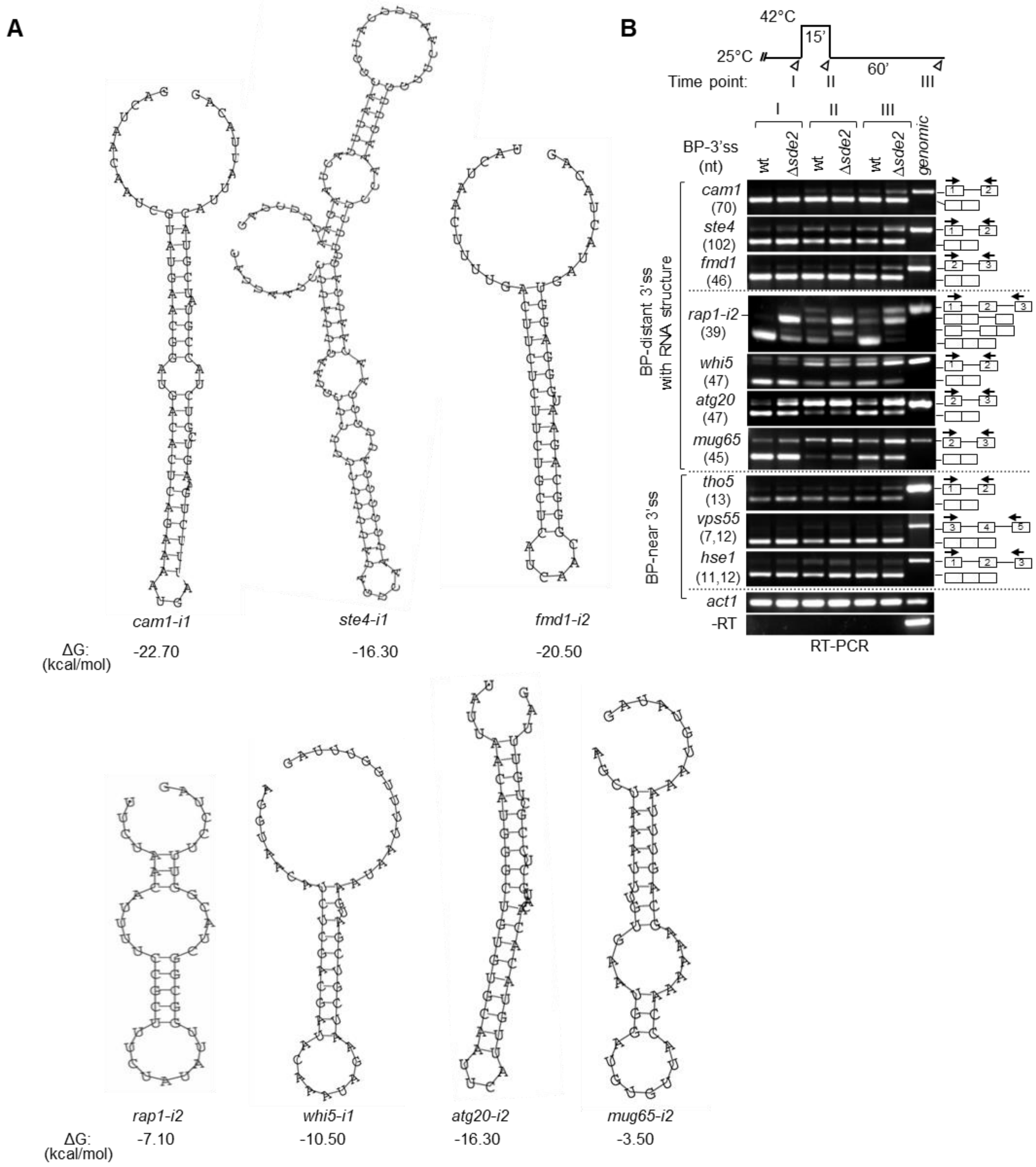

Figure S7. RNA structures bring 3'ss closer to BP.

(A) Predicted secondary structure of the RNA between BP and 3'ss in different introns. ΔG refers to the Gibbs free energy change in kcal/mol.

(B) Temperature treatment regime and splicing assay by RT-PCR. The open arrowheads show the time-point of the harvest. Semiquantitative RT-PCRs for BP-near 3'ss and BP-distant 3'ss introns with RNA structures to monitor the splicing. Prolonged treatment at 42°C or higher temperature leads to general splicing defects. Numbers in parentheses indicate the distance between BP and 3'ss. The dotted lines group genes with respect to the heat sensitivity of their splicing.

Protein product, Systematic ID Intron number	BP-3’ss (nt)	log_2 intron retention ratio *Δsde2*/wt at 30°C	log_2 intron retention ratio *Δsde2*/wt at 37°C	Structure predicted by RNAfold web server	ΔG (Kcal/mol)
DNA helicase in rearranged telomeric region, SPAC212.06c Intron-1	26	2.47	2.38	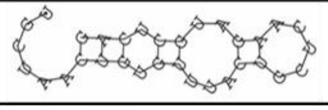	-2.2
Hif2, Set3 complex subunit, SPCC1235.09 Intron-1	23	1.36	0.762	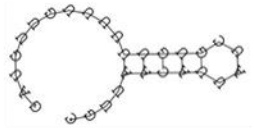	-1.1
Rap1, shelterin complex telomere binding subunit, SPBC1778.02 Intron-2	39	1.32	1.35	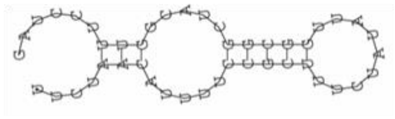	-7.1
Sterol intermembrane transfer protein, SPBC354.07c Intron-2	28	1.31	1.32	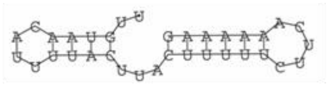	-0.9
Psf3, GINS complex subunit, SPAC227.16c Intron-4	21	1.30	1.33	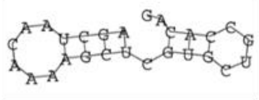	-3.2
Heat shock factor binding protein, SPBC16E9.15 Intron-3	22	1.29	1.18	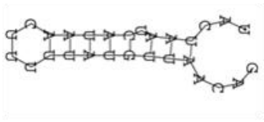	-1.1
Ubc11, ubiquitin conjugating enzyme E2, SPCC1259.15c Intron-2	18	1.19	0.89	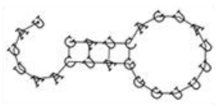	-2
Pdx1, pyruvate dehydrogenase complex SPCC1259.09c Intron-1	18	1.17	0.82	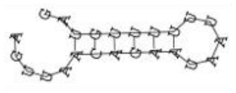	-3.1
Ftp105, Golgi localized, SPAC17A5.16 Intron-3	24	1.14	0.94	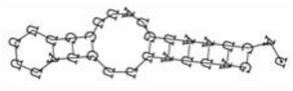	-2.3
Apc10, anaphase promoting complex substrate recognition subunit, SPBC1E8.06 Intron-2	11	1.02	1.14	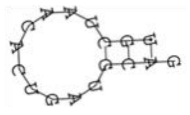	-1.4
Dcd1, deoxycytidylate deaminase SPBC2G2.13c Intron-1	36	1.02	0.61	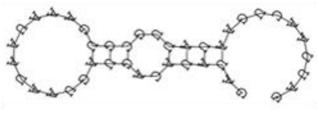	-0.3

Figure S8. ***rap1*** **intron-2 -like Sde2-dependent introns with BP-distant 3’ss and the intervening RNA structured**

Predicted secondary structure of Sde2-dependent introns in decreasing order of splicing defect in *Δsde2* strain (log_2 intron retention ratio *Δsde2*/wt at 30°C). The secondary structure was predicted using an RNAfold web server (2). ΔG refers to the Gibbs free energy change in kcal/mol.

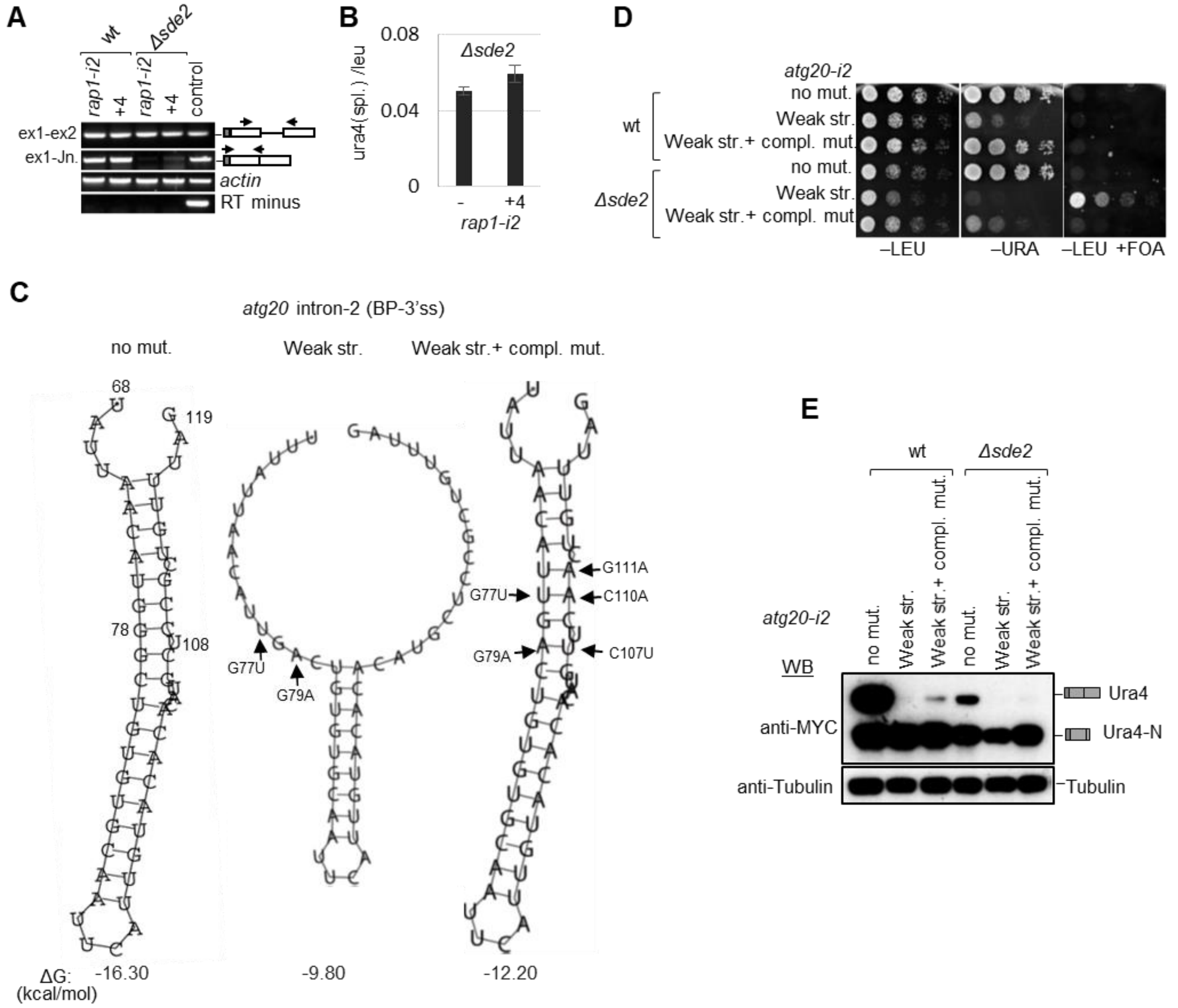

Figure S9. RNA structures bring 3'ss closer to BP.

(A) Semiquantitative RT-PCR shows the splicing of *rap-i2* variants with different secondary structures. RT-PCR was performed in the same way as in Figure 2D.

(B) qRT-PCR for secondary structure strengthened *rap1-i2*. (A) and (B) are an extension of Figure 5E-F

(C) Predicted secondary structure of RNA between BP and 3'ss in three variants of *atg20-i2.* ΔG refers to the Gibbs free energy change in kcal/mol. Arrows indicate the position of mutations with respect to the 'no mut' construct of *atg20-i2.*

(D) The growth assay indicates splicing of a*tg20-i2* with a different secondary structure between BP and 3'ss (incubation time: -LEU - 3 days, -URA, +FOA - 4 days).

(E) Immunoblot analysis to monitor a*tg20-i2* splicing with different secondary structures.

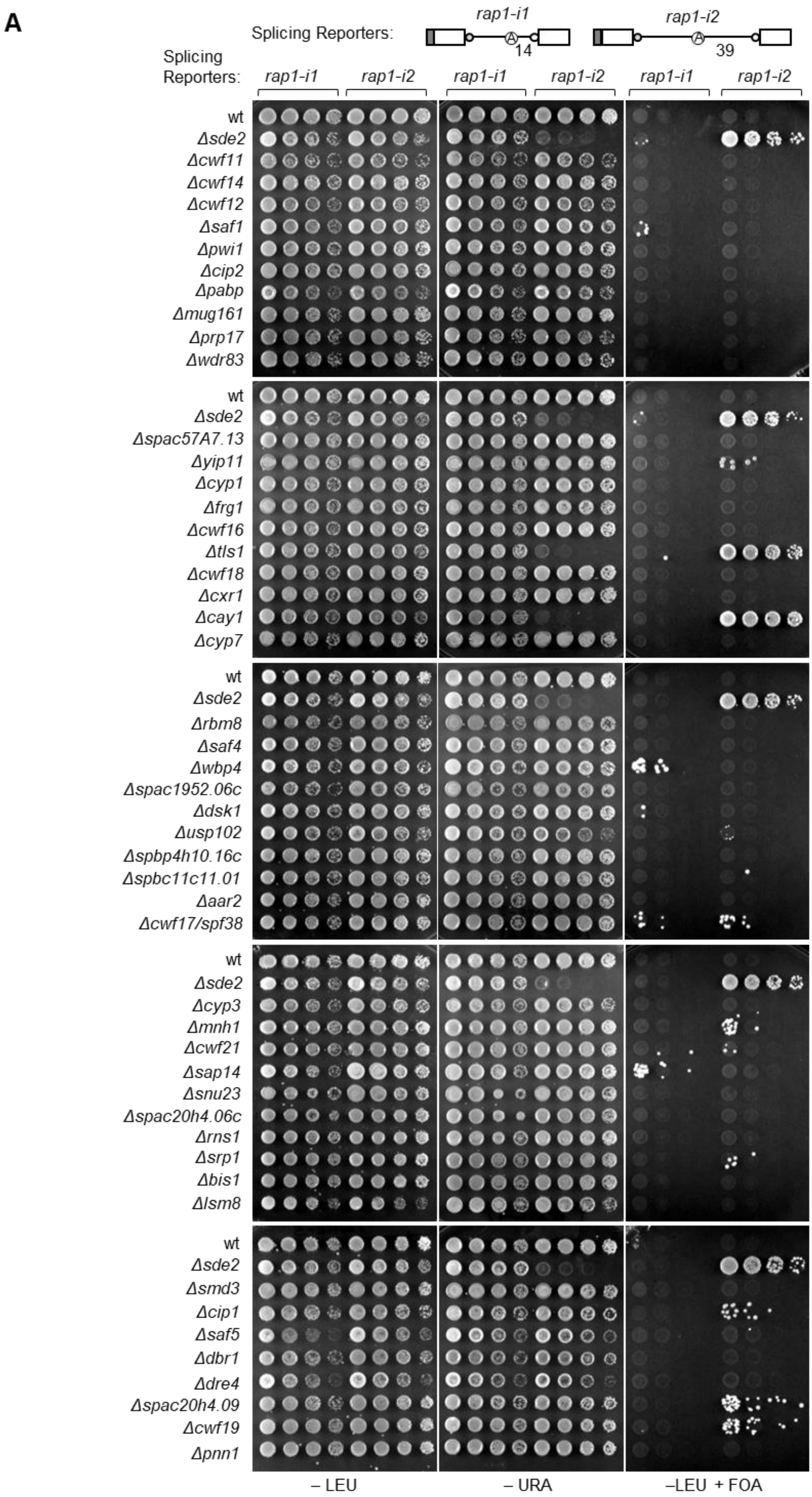

Figure S10. Search for additional Sde2-like intron-specific splicing factors.

(A) The growth of different viable splicing factor deletion strains was checked with the *rap1-i1* and *rap1-i2* reporter (incubation time: -LEU, -URA - 4 days; +FOA - 5 days).

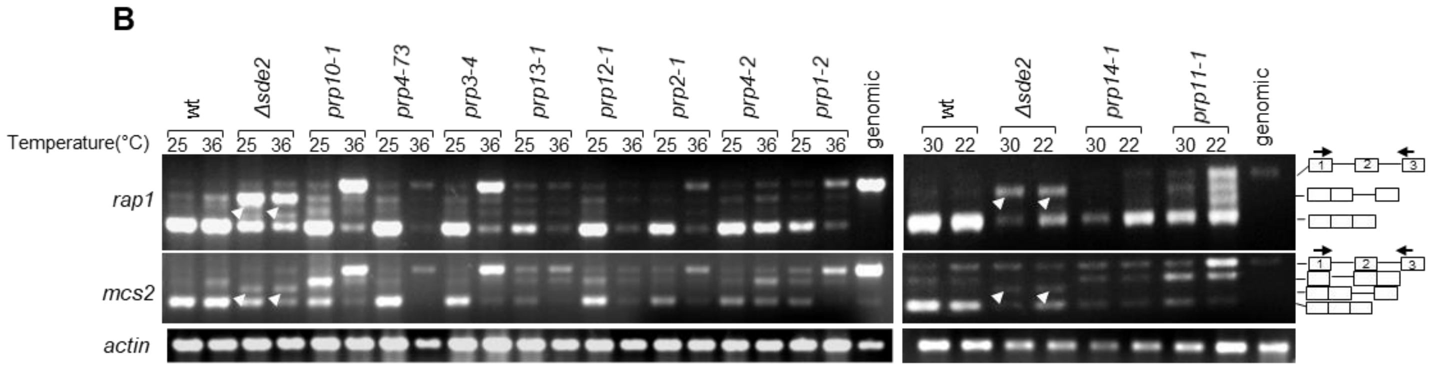

Figure S10. (cont.).

(B) Semiquantitative RT-PCR was performed in the indicated strains for selected targets. All the PCRs were run on independent gels. Arrows indicate where the primer binds in the schematics, the number in the blocks indicates exon number, and white arrowheads indicate introns dependent on Sde2. *S. pombe* genomic DNA was used as a control to size intron-containing transcript. Temperature-sensitive strains were kept at 36°C for 3 hrs, and cold-sensitive strains were kept at 22°C for 6 hrs.

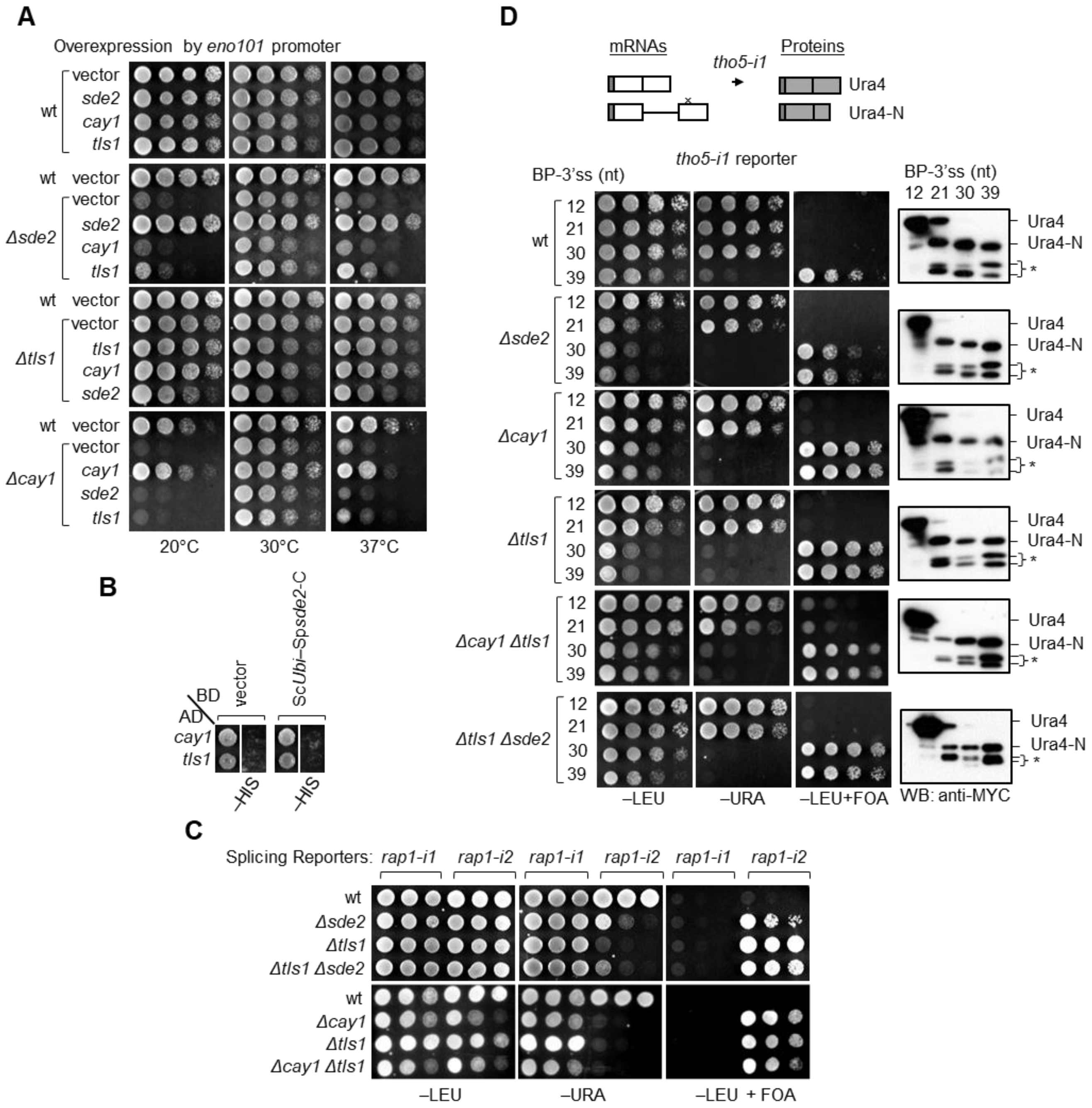

Figure S11. Interaction among intron-specific splicing factors.

(A) Overexpression constructs were expressed in deletion strains grown at different temperatures (incubation time: 30ºC, 37ºC - 3 days; 20ºC - 5 days).

(B) Yeast two-hybrid did not show any interaction between Sde2, Cay1, and Tls1.

(C) Double mutants showed splicing defects similar to single deletions for the *rap1-i1* and *rap1-i2* reporters (incubation time: -LEU, -URA - 4 days; +FOA - 5 days).

(D) Growth assay and immunoblot analysis indicate that the increase in distance between BP and 3'ss led to splicing defects in double mutants similar to the single mutants (For wt, *Δsde2*, *Δcay1*, and *Δtls1*-incubation time: -LEU - 3days, -URA - 4 days; +FOA - 4 days; For *Δcay1 Δtls1 and Δsde2*, *Δtls1*- incubation time: -LEU, -URA - 4 days; +FOA - 5 days).

A

Before stop codon		From stop codon	
Gene	Segment (bp)	Gene	Segment (bp)
Rap1	HindIII-431-SalI	Rap1	EcoRI-532-SpeI
Bqt3	HindIII-511-SalI	Bqt3	EcoRI-542-SpeI
Pst2	HindIII-541-SalI	Pst2	EcoRI-357-SpeI
Hif2	HindIII-453-SalI	Hif2	EcoRI-524-SpeI
Rxt2	SalI-425-BamHI	Rxt2	EcoRI-398-SpeI
Dsh1	HindIII-480-SalI	Dsh1	EcoRI-488-SpeI

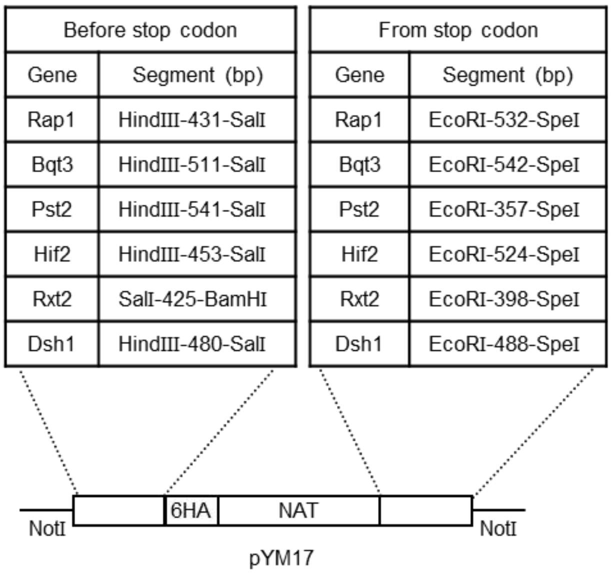

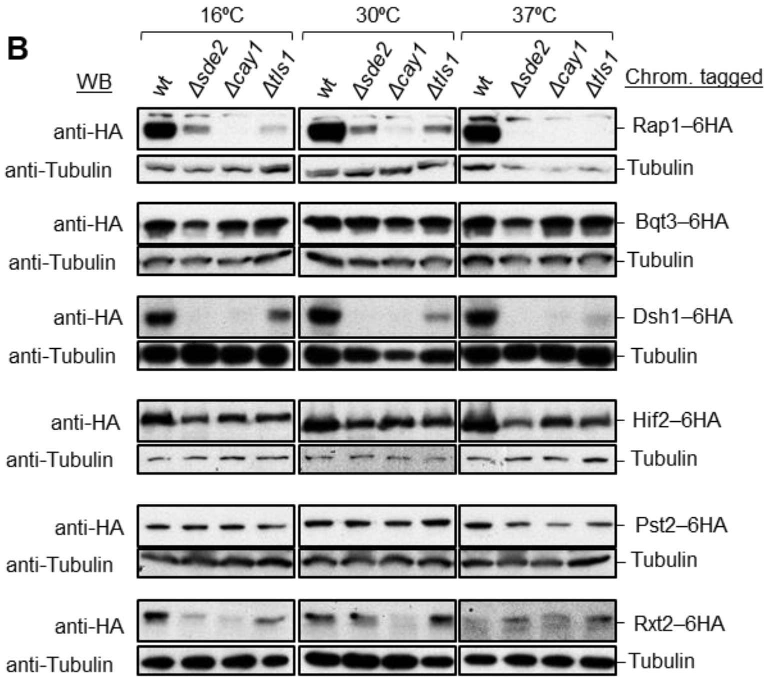

Figure S12. Sde2, Cay1, and Tls1 control the expression of chromatin factors.

(A) Schematic shows the design of constructs to chromosomally tag indicated genes with 6HA epitope tags at their C-termini.

(B) Immunoblot analysis of indicated heterochromatin factors (Rap1, Bqt3, Dsh1, Hif2, Psf3, and Rxt2) shows reduced levels in mutants. The liquid cultures were treated for 3 hrs at 37°C or 8 hrs at 16°C.

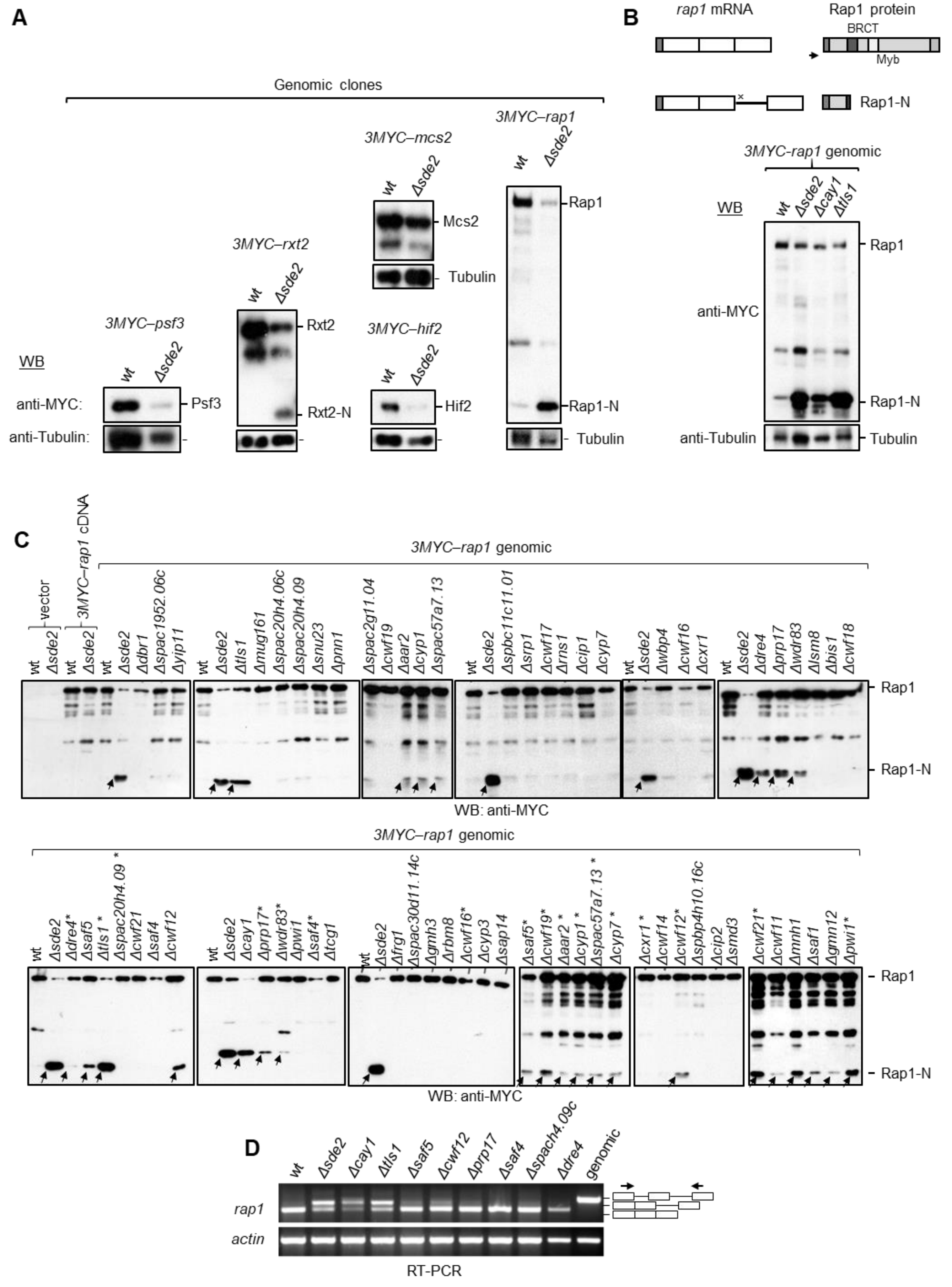

Figure S13. Search to monitor the expression of the alternatively spliced form (Figure legends on next page).

(A) Protein analysis shows Mcs2, Hif2, Psf3, Rxt2, and Rap1 proteins in *Δsde2* deletion strain compared to the wt strain. Both Rap1 and Rxt2 proteins showed alternative forms in *Δsde2* strain. Genomic constructs were expressed under the *nmt81* promoter.

(B) An alternative form of the Rap1 protein (Rap1-N) accumulated in the deletion mutants. The genomic construct of Rap1 under the *eno101* promoter was expressed in the mutants.

(C) Search to monitor Rap1-N expression in different viable splicing factor deletion mutants. The N-terminal 3MYC-tagged *rap1* genomic construct under the *eno101* promoter was expressed in the deletion mutants of putative 50 splicing factors (from the Bioneer *S. pombe* haploid deletion library) and alternatively spliced form of Rap1 protein (Rap1-N) was monitored by anti-MYC western blots. The following deletion strains showed accumulation of Rap1-N: *Δdre4*, *Δprp17*, *Δwdr83*, *Δsaf5*, *Δcwf12*, *Δcwf21*, *Δcwf19*, *Δaar2*, *Δcyp1*, *Δspac57a7.13*, *Δpwi1*, *Δsaf1*, *Δgmi12*, *Δcwf11*, *Δcyp7*, *Δmnh1*. Arrows indicate mutants showing the presence of Rap1-N. Asterisk shows a repeat of the analysis.

(D) Semiquantitative PCR to monitor Rap1 splicing in some of the factors obtained from the search.

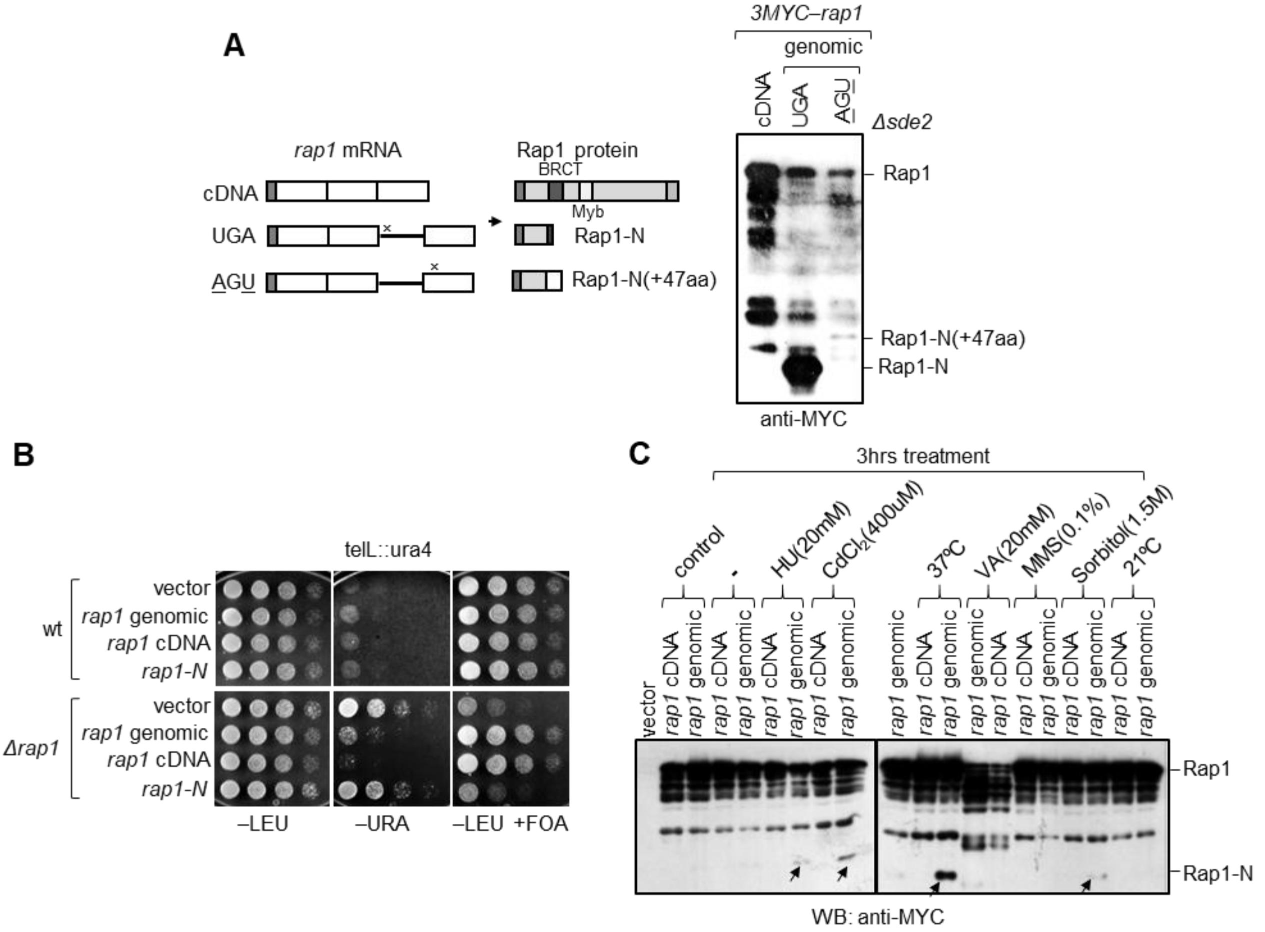

Figure S14. The physiological relevance of the alternatively spliced form of Rap1 (Rap1-N).

(A) Rap1-N is formed due to the retention of intron-2. × marks the stop codon arising during the translation of the intron-retained mRNA. Western blot with the stop codon mutated intron-2 version of *rap1* genomic construct. Instead of Rap1-N, an upshifted band was observed because of a new stop codon in exon 2.

(B) wt and *Δrap1* telomeric reporter strains expressing *rap1* variants (genomic, cDNA and *rap1-N*) under their promoter (incubation time: -LEU, -URA - 3 days; +FOA - 5 days).

(C) Rap1-N accumulated under stress conditions. wt cells transformed with different constructs of *rap1* (genomic and cDNA) were treated for 3 hours, and immunoblotting was performed using an anti-MYC antibody. The constructs were expressed under the *eno101* promoter. Arrows indicate samples that show the presence of Rap1-N (HU, Hydroxyurea; $CdCl_2$, Cadmium chloride; VA, Valproic acid; MMS, Methyl methanesulfonate).

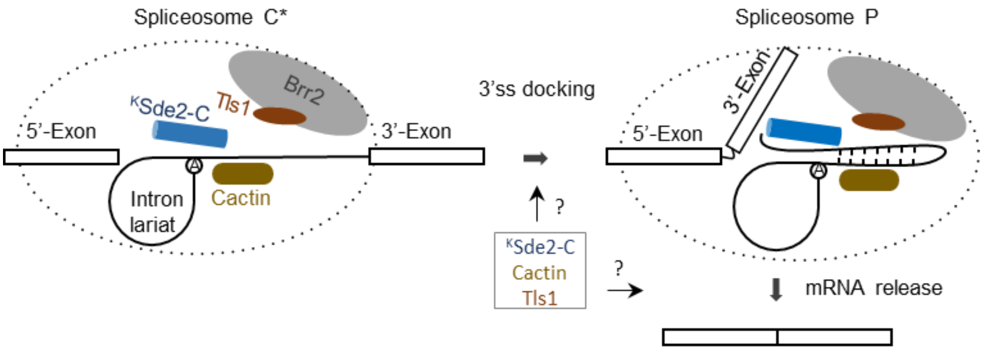

Figure S15. Proposed mechanism of intron-specific splicing factors Sde2, Cactin, and Tls1.

Sde2 is shown to be present in the C* complex in the cryo-EM structure of the human spliceosome. Sde2 recruits Cactin into the spliceosome (3, 4). Tls1 interacts with Brr2, a U5 snRNP specific RNA helicase, in humans and *S. pombe* (5, 6). Here, we propose that Sde2 recruits Cactin, stabilising the RNA between BP and 3'ss and therefore bridging the gap between BP and 3'ss. Tls1, on the other hand, might regulate Brr2's activity which could be important for splicing the BP-distant 3'ss introns. These factors can also play a role as mRNA release factors.

Table S1. Features of Sde2 target introns: Top targets of Sde2. Extension of the table EV2 (Features of sde2 target introns) with the added distance between BP and 3'ss (3) ('-'not able to detect BP in this intron). Underline represents nucleotides that differ from the canonical splice sites. Targets in bold represent BP-near 3'ss (BP-3'ss distance ≤12 nt).

Gene name (Systematic ID from PomBase)	Sde2-target intron (total no.)	Length (nt)	5' Splice sites (canonical = GTAAGT)	3' Splice sites (canonical = TAG)	Predicted branch points (canonical = CTAAC)	Distance between BP and 3'ss
SPAC212.06C	1(1)	42	GTGCAT	CAG	CTAAC	26
SPAC56E4.08C	1(1)	45	GTAATG	TAG	TTAAC	15
paa1 (SPAP8A3.09C)	1(6)	51	GTGTGT	TAG	TTAAC	16
gga22 (SPBC25H2.16C)	2(5)	53	GTAGGA	TAG	CTAAC	19
hif2 (SPCC1235.09)	1(3)	90	GTATGT	TAG	TTAAC	23
rap1 (SPBC1778.02)	2(2)	83	GTATGA	TAG	CTAAC	39
SPBC354.07C	2(4)	122	GTATGA	AAG	CTAAC/ GTAAC	28
psf3 (SPAC227.16C)	4(4)	59	GTATGT	CAG	CTAAC	21
SPBC16E9.15	3(3)	64	GTACGA	CAG	ATAAC/CTAAT	16/22
**SPAC16A10.03C**	**2(3)**	**43**	**GTACGT**	**TAG**	**CTAAC**	**11**
rga4 (SPBC28E12.03)	2(3)	142	GTATGA	TAG	TTGAT/CTAAT	17
mcs2 (SPBP16F5.02)	2(2)	52	GTATGT	TAG	CTAAC	20
ptl2 (SPAC31G5.20C)	1(3)	83	GTAGGA	TAG	TTAAC	25
ubc11 (SPCC1259.15C)	2(4)	50	GTTTGT	TAG	TTAAC	18
apc10 (SPBC1E8.06)	1(2)	71	GTAAGT	AAG	CTAAC	23
**apc10 (SPBC1E8.06)**	**2(2)**	**58**	**GTACGT**	**CAG**	**TTAAT/CTAAC**	**11**
pdx1 (SPCC1259.09C)	1(5)	59	GTGAGT	TAG	TTAAC/ATAAC	18
bqt3 (SPCC594.07c)	1(1)	49	GTATGC	AAG	CTAAC	14
ftp105 (SPAC17A5.16)	3(7)	68	GTATGA	TAG	CTAAT	24
vid21 (SPCC1795.08c)	1(2)	82	GTACGT	AAG	CTAAT/TTAAT	21
kap114 (SPAC22H10.03C)	1(6)	56	GTATGC	AAG	CTAAC	15
dcd1 (SPBC2G2.13C)	1(3)	192	GTATGT	TAG	CTAAC	36
pex7 (SPAC1834.12)	2(2)	129	GTAAGT	TAG	CTAAC	13
naa20 (SPCC16C4.12)	1(1)	57	GTAAGT	TAG	TTAAC	16
dom34 (SPCC18B5.06)	2(4)	60	GTATGA	TAG	ATAAC	23
SPBC660.16	4(4)	172	GTAATG	CAG	CTAAC	17
**mmp2 (SPBC336.13c)**	**2(2)**	**67**	**GTAGGT**	**CAG**	**TTAAC**	**11**
rxt2 (SPBC428.06C)	4(4)	180	GTAAGT	TAG	CTAAC	17
ypt5 (SPAC6F6.15)	3(7)	76	GTATGC	CAG	CTGAC/TTAAT	17/35
**atp10 (SPAC4G8.11c)**	**2(2)**	**101**	**GTAAGT**	**CAG**	**ATAAC/CTAAC**	**7**
pre10 (SPCC1795.04C)	3(3)	66	GTACGT	TAG	CTAAT	18
pku70 (SPCC126.02c)	3(5)	74	GTAAAA	AAG	CTGAT/CTAAT	19
vma5 (SPAPB2B4.05)	3(6)	52	GTATGT	CAG	CTAAC	16
plp2 (SPBC2A9.09)	2(2)	63	GTACGT	AAG	CTAAC	16
eaf1 (SPCC1223.10C)	1(3)	102	GTACGT	TAG	-	-
**tbp1 (SPAC29E6.08)**	**2(3)**	**52**	**GTACGT**	**TAG**	**TTGAC**	**10**

Table S2. Plasmids used in this study.

**Plasmid name**	**Description**	**Ref.**
Peno101-3MYC–sde2–3FLAG	*S. pombe sde2* gene under *eno101* promoter with 3MYC tag at the N-terminus and 3FLAG tag at the C-terminus	(3)
pREP81x-3MYC–rap1 cDNA	*S. pombe rap1* cDNA in pREP81x with 3MYC tag at the N-terminus	This study
pREP81x-3MYC–rap1 genomic	*S. pombe rap1* genomic sequence in pREP81x with 3MYC tag at the N-terminus	This study
pREP81x-3MYC–rap1 genomic (stop codon removed)	*S. pombe rap1* genomic sequence where 5' splice site of intron-2 is mutated to GTAAGT in pREP81x	This study
pREP81x-3MYC–psf3 genomic–3FLAG	*S. pombe psf3* genomic sequence in pREP81x with 3MYC tag at the N-terminus and 3FLAG tag at the C-terminus	This study
pREP81x-3MYC–mcs2 genomic–3FLAG	*S. pombe mcs2* genomic sequence in pREP81x with 3MYC tag at the N-terminus and 3FLAG tag at the C-terminus	This study
pREP81x-3MYC–hif2 genomic–3FLAG	*S. pombe hif2* genomic sequence in pREP81x with 3MYC tag at the N-terminus and 3FLAG tag at the C-terminus	This study
pREP81x-3MYC–rxt2 genomic–3FLAG	*S. pombe rxt2* genomic sequence in pREP81x with 3MYC tag at the N-terminus and 3FLAG tag at the C-terminus	This study
Peno101-3MYC–rap1 genomic	*S. pombe* rap1 genomic sequence under *eno101* promoter with 3MYC tag at the N-terminus	This study
Peno101-3MYC–rap1 cDNA	*S. pombe* rap1 cDNA under *eno101* promoter with 3MYC tag at the N-terminus	This study
Prap1-3MYC–rap1 genomic	*S. pombe rap1* genomic sequence under *rap1* 1000bp promoter with 3MYC tag at the N-terminus	This study
Prap1-3MYC–rap1 cDNA	*S. pombe rap1* cDNA under *rap1* 1000bp promoter with 3MYC tag at the N-terminus	This study
Prap1-3MYC–rap1-N	*S. pombe rap1 genomic sequence till the stop codon in intron-2 under 1000bp rap1 promoter with 3MYC tag at the N-terminus*	This study
Prap1-3MYC–rap1 cDNA (ura4+)	*S. pombe rap1 cDNA under 1000bp rap1 promoter with 3MYC tag at the N-terminus in pREP82x vector*	This study
Peno101-3MYC–cay1–3FLAG	*S. pombe cay1 cDNA under eno101 promoter with 3MYC tag at the N-terminus and 3FLAG tag at the C-terminus*	This study
Peno101-tls1	*S. pombe tls1 under eno101 promoter*	This study
pYM17 rap1–6HA	The plasmid used to insert 6HA at the C-terminus of the *rap1* gene chromosomally. See schematic in supplementary Figure 12a	This study
pYM17 bqt3–6HA	The plasmid used to insert 6HA at the C-terminus of the *bqt3* gene chromosomally. See schematic in supplementary Figure 12a	This study
pYM17 pst2–6HA	The plasmid used to insert 6HA at the C-terminus of the *pst2* gene chromosomally. See schematic in supplementary Figure 12a	This study
pYM17 hif2–6HA	The plasmid used to insert 6HA at the C-terminus of the *hif2* gene chromosomally. See schematic in supplementary Figure 12a	This study
pYM17 rxt2–6HA	The plasmid used to insert 6HA at the C-terminus of the *rxt2* gene chromosomally. See schematic in supplementary Figure 12a	This study

pYM17 dsh1–6HA	The plasmid used to insert 6HA at the C-terminus of the *dsh1* gene chromosomally. See schematic in supplementary Figure 12a	This study
pGBDC1 ScUbi1–Spsde2C	*S. pombe sde2* with its UBL domain replaced with *S. cerevisiae* ubiquitin in the pGBDU-C1 vector	This study
pGADC1-cay1	*S. pombe cay1* cloned in the pGAD-C1 vector	This study
pGADC1-tls1	*S. pombe tls1* cloned in the pGAD-C1 vector	This study

Table S3. *S. pombe* strains used in this study.

Strain ID	Genotype	Ref.
JY741	*h– ade6-M216 leu1 ura4-D18*	
JY746	*h– ade6-M216 leu1 ura4-D18*	
SP20	*h+ Δsde2::Nat-NT2*	(3)
SP38	*h– sde2–6HA Nat-NT2*	(3)
SP52	*h+ h+ Δubp15::KanMX4, Δubp5::Nat-NT2*	(3)
SP68	*h+ leu1-32 ura4DS/E his3 ade6-D1 ade6-M210 otr1R::ade6+ tel1L::his3+ tel2L::ura4+ Δsde2Δ::kan*	NBRP
SP77	*h– sde2::sde2(AAK)–6HA Nat-NT2*	(3)
SP82	*h+ sde2::sde2(K85M)–6HA Nat-NT2*	(3)
SP140	*h– rap1–6HA Nat-NT2*	This study
SP141	*h– bqt3–6HA Nat-NT2*	This study
SP142	*h– pst2–6HA Nat-NT2*	This study
SP143	*h– hif2–6HA Nat-NT2*	This study
SP144	*h– rxt2–6HA Nat-NT2*	This study
SP145	*h– dsh1–6HA Nat-NT2*	This study
SP146	*h+ Δsde2::KanMX4 rap1–6HA Nat-NT2*	This study
SP147	*h+ Δsde2::KanMX4 bqt3–6HA Nat-NT2*	This study
SP148	*h+ Δsde2::KanMX4 pst2–6HA Nat-NT2*	This study
SP149	*h+ Δsde2::KanMX4 hif2–6HA Nat-NT2*	This study
SP150	*h+ Δsde2::KanMX4 rxt2–6HA Nat-NT2*	This study
SP151	*h+ Δsde2::KanMX4 dsh1–6HA Nat-NT2*	This study
SP152	*h+ Δcay1::KanMX4 rap1–6HA Nat-NT2*	This study
SP153	*h+ Δcay1::KanMX4 bqt3–6HA Nat-NT2*	This study
SP154	*h+ Δcay1::KanMX4 pst2–6HA Nat-NT2*	This study
SP155	*h+ Δcay1::KanMX4 hif2–6HA Nat-NT2*	This study
SP156	*h+ Δcay1::KanMX4 rxt2–6HA NAT-NT2*	This study
SP157	*h+ Δcay1::KanMX4 dsh1-6HA Nat-NT2*	This study
SP158	*h+ Δtls1::KanMX4 rap1–6HA Nat-NT2*	This study
SP159	*h+ Δtls1::KanMX4 bqt3–6HA Nat-NT2*	This study
SP160	*h+ Δtls1::KanMX4 pst2–6HA Nat-NT2*	This study

SP161	*h+ Δtls1::KanMX4 hif2–6HA Nat-NT2*	This study
SP162	*h+ Δtls1::KanMX4 rxt2–6HA Nat-NT2*	This study
SP163	*h+ Δtls1::KanMX4 dsh1–6HA Nat-NT2*	This study
SP164	*h+ Δtls1::KanMX4 Δsde2::Nat-NT2*	This study
SP166	*h+ leu1-32 ura4DS/E his3 ade6-D1 ade6-M210 otr1R::ade6+ tel1L::his3+ tel2L::ura4+ Δrap1::Nat-NT2*	This study
SP188	*h+ sde2::ubi–sde2-C–6HA Nat-NT2*	This study
SP192	*h+ Δtls1::KanMX4 Δcay1::Nat-NT2*	This study
SP193	*h+ leu1-32 ura4DS/E his3 ade6-D1 ade6-M210 otr1R::ade6+ tel1L::his3+ tel2L::ura4+*	This study
SP121	*h- prp10-1 otr1Rsph1::ade6 lys1::Nat ade6-DN/N leu1-32 ura4-D18/DS/E*	Gift from Robin Allshire
SP124	*h- leu1-32 ura4-D18 prp4-73*	NBRP
SP127	*h- prp3-4*	NBRP
SP128	*h- prp13-1*	NBRP
SP129	*h-prp12-1*	NBRP
SP132	*h- prp2-1 leu1-32*	NBRP
SP170	*h+ prp4-2 ade6-216*	NBRP
SP171	*h- prp1-4 leu1-32*	NBRP
SP130	*prp14-1*	NBRP
SP131	*prp11-1*	NBRP

Table S4. *ura4* splicing reporters used in this study.

Splicing Reporters	Introns inserted in *ura4* gene (BP is shown in bold case letters, mutations are underlined)	Nucleotides between BP and 3'ss
Peno-3MYC–ura4	*ura4* ORF under *eno101* promoter without intron	
rap1-i1	GTATGGATTAATCATTATCAAAAAATTT**CTAAT**CATTATTATTTAG	14
rap1-i2	GTATGATCTTGCTTACCATTAATTGTTTTTTATTTTTTTT**CTAAC**ATTTTCC GCTTTCTATATTGGCGGCTACGGTTTCCTAG	39
tho5-i1	GTACGTGAAAGTTCCTTACCTTTTTTTTTTTTTTTTTCAGATTTCAAAAGTC TTTGTTCATTTCTTT**CTAAC**CATTTTAATAG	12
tho5-i1 (BP mutant)	GTACGTGAAAGTTCCTTACCTTTTTTTTTTTTTTTTTCAGATTTCAAAAGTC TTTGTTCATTTCTTT**TTAAC**CATTTTAATAG	12
tho5-i1 (BP mutant)	GTACGTGAAAGTTCCTTACCTTTTTTTTTTTTTTTTTCAGATTTCAAAAGTC TTTGTTCATTTCTTT**CTAAA**CATTTTAATAG	12
tho5-i1 (3'ss mutant)	GTACGTGAAAGTTCCTTACCTTTTTTTTTTTTTTTTTCAGATTTCAAAAGTC TTTGTTCATTTCTTT**CTAAC**CATTTTAAAAG	12
rap1-i2 (BP-3'ss dist. 12 nt)	GTATGATCTTGCTTACCATTAATTGTTTTTTATTTTTTTTCTTTCATTTTCC GCTTTCTATATTGTT**CTAAC**CGGTTTCCTAG	12
rap1-i2 (5'ss-BP dist. 70 nt)	GTATGATCTTGCTTACCATTAATTGTTTTTTATTTTTTAGATTTCAAAAGTC TTTGTTCATTTCTTT**CTAAC**ATTTTCCGCTTTCTATATTGGCGGCTACGGT TTCCTAG	39

pyp3-i1 (BP-3'ss dist. 23 nt)	GTTAGGAATAAAAAGAAATTGCGGGATTGTAAACGGCTTTGGCCTTTTTTCTTTTCATCATTTTTTGTAACATAATTA**CTAAC**TTAGTCTTCTTTATTTATATAG	23
ftp105-i3 (BP-3'ss dist. 24 nt)	GTATGAAATTTTTTTTATTAACTTGTGTAAGACGCTTTTA**CTAAT**GTATTCGTTTTTATGTTTATTAG	24
ftp105-i3 (BP-3'ss dist. 12 nt)	GTATGAAATTTTTTTTATTAACTTGTGTAAGACGCTTTTACTATTGTATTCG**CTAAT**ATGTTTATTAG	12
tho5-i1 (BP-3'ss dist. 39 nt)	GTACGTGAAAGTTCCTTACCTTTTTTTTTTTTTTTTTCTT**CTAAC**AAAATTCTTTGTTCATTTCTTTCTTTCCATTTTAATAG	39
tho5-i1 (BP-3'ss dist. 30 nt)	GTACGTGAAAGTTCCTTACCTTTTTTTTTTTTTTTTTCAGATTTCAATT**CTAAC**TGTTCATTTCTTTCTTTCCATTTTAATAG	30
tho5-i1 (BP-3'ss dist. 21 nt)	GTACGTGAAAGTTCCTTACCTTTTTTTTTTTTTTTTTCAGATTTCAAAAGTCTTTGTT**CTAAC**CTTTCTTTCCATTTTAATAG	21
tho5-i1 (BP-3'ss dist. 16 nt)	GTACGTGAAAGTTCCTTACCTTTTTTTTTTTTTTTTTCAGATTTCAAAAGTCTTTGTTCATTTCT**CTAAC**TATCCATTTTAATAG	16
tho5-i1 (BP-3'ss dist. 9 nt)	GTACGTGAAAGTTCCTTACCTTTTTTTTTTTTTTTTTCAGATTTCAAAAGTCTTTGTTCATTTCTTTCTA**CTAAC**TTTAATAG	9
tho5-i1 (BP-3'ss dist. 6 nt)	GTACGTGAAAGTTCCTTACCTTTTTTTTTTTTTTTTTCAGATTTCAAAAGTCTTTGTTCATTTCTTTCTAGCC**CTAAC**AATAG	6
rap1-i2 C52G C54G , (i)	GTATGATCTTGCTTACCATTAATTGTTTTTTATTTTTTTT**CTAAC**ATTTTCGGGTTTCTATATTGGCGGCTACGGTTTCCTAG	39
rap1-i2 G66C G68C, (ii)	GTATGATCTTGCTTACCATTAATTGTTTTTTATTTTTTTT**CTAAC**ATTTTCCGCTTTCTATATTGCCCGCTACGGTTTCCTAG	39
rap1-i2 C52G C54G G66C G68C, (i + ii)	GTATGATCTTGCTTACCATTAATTGTTTTTTATTTTTTTT**CTAAC**ATTTTCGGGTTTCTATATTGCCCGCTACGGTTTCCTAG	39
rap1-i2 (+1)	GTATGATCTTGCTTACCATTAATTGTTTTTTATTTTTTTT**CTAAC**CTTTTCCGCTTTCTATATTGGCGGCTACGGTTTCCTAG	39
rap1-i2 (+3)	GTATGATCTTGCTTACCATTAATTGTTTTTTATTTTTTTT**CTAAC**ATTTTCCGCTTTCTATATGGGCGGAAACGGTTTCCTAG	39
rap1-i2 (+4)	GTATGATCTTGCTTACCATTAATTGTTTTTTATTTTTTTT**CTAAC**ATTTTCCGCTTTCTATATTGGCGGCGGCGAATTCCTAG	39
atg20-i2	GTAAGTTTTCCAATATATTTAGATATAATATCTTTCATCGCGTTTTTTCATTTTCTTTTCAAACGTTTA**TTAAC**ATGGGCTGTGTGCAATTCATTGTACACACATGCTCCGCTGTTTAG	46
atg20-i2 (weak structure)	GTAAGTTTTCCAATATATTTAGATATAATATCTTTCATCGCGTTTTTTCATTTTCTTTTCAAACGTTTA**TTAAC**ATTGACTGTGTGCAATTCATTGTACACACATGCTCCGCTGTTTAG	46
atg20-i2 (weak structure + compl.)	GTAAGTTTTCCAATATATTTAGATATAATATCTTTCATCGCGTTTTTTCATTTTCTTTTCAAACGTTTA**TTAAC**ATTGACTGTGTGCAATTCATTGTACACACATGTTCAACTGTTTAG	46
tho5-i1 (competing 3'ss)	GTACGTGAAAGTTCCTTACCTTTTTTTTTTTTTTTTTCAGATTTCAAAAGTCTTTGTT**CTAAC**CTTTCTTTTAGTTTTAATAG	12, 21

Table S5. Primers used for RT-PCR assays.

Primer name	Primer sequence (5’-3’)
rap1 Ex1 F	CCAAAAGCGATGGCTCGTCC
rap1 Ex3 R	AACCGAAGCAGACTTGGAAATC
act1 F	CCCCTAGAGCTGTATTCCC
act1 R	CCAGTGGTACGACCAGAGG
ura R	CTGTGTAGGAACCAGTAGCC
ura F	GTACAAAATTGCTTCTTGG
ura junction R	AGAGACCACGTCCCAAAG
tef 3’UTR R	CTTTTACCCGGGTTAATGCTG
LEU2 F	CTGATACCTGCATCCAAAACC
LEU2 R	ACCGTTCCAGAAGTGCAAAG
myc F	CTCAGAAGAAGACTTGAACGGATCCATG
tho5 Ex1 F	CGATAACGCCCGTATACGGC
tho5 Ex2 R	CTGCTTTAAGGTGGAAAGTCG
vps55 Ex3 F	TATGCTGGTGATCCTGTCTTG
vps55 Ex5 R	CCGGTACAGATGGTTGCACC
hse1 Ex1 F	ATGTTTCGAGGAAAACCCAAC
hse1 Ex3 R	GAGTCACTAGCTATTTTCAAAAGAG
whi5 Ex1 F	GCTACAAGGCAGACTGATGAAGT
whi5 Ex2 R	CTGATCTTCCCATCCATGATCGACC
atg20 Ex2 F	TCCGTTTGGCGGAGATATTC
atg20 Ex3 R	GGATGCAAAAGACACTGACG
mug65 Ex2 F	GGGTTGATCAGCATTTTGTTGCG
mug65 Ex3 R	CGTCTGCGACGAACAAACG
ste4 Ex1 F	GGAATTGGAACAATGAAGCAGTTTG
ste4 Ex2 R	CCTTCTGCTGCTTTTTCATTGACTG
cam1 Ex1 F	GAAGGACTTTAGAGTTACTGATTTTTACC
cam1 Ex2 R	CTTGTAATTCGGCGGCAGTAGG
fmd1 Ex2 F	GCTTGGGGTGCAAAGGAACC
fmd1 Ex3 R	CCAAGAACAATAGGGAATGCGCC
mcs2 Ex1 F	GCACTTTCTTCCGCTCTTTCC
mcs2 Ex3 R	TAATGATTGACAAACGTTAAATTCG
psf3 Ex3 F	GTATCTATTCGGGACATAACCACAC
psf3 Ex5 R	CTACGAAGTGGAATTTTGCC
hif2 Ex1 F	CTGGAGATATTTGAAAGAATGC
hif2 Ex2 R	AGCTTTCGTCAGTGTCG
dsh1 Ex1 F	GGCAGAAAATAAGAAATTTTC
dsh1 Ex1 5	TTTTACACTGTCGCATCG
tho5 i1 lariat	GGTAAGGAACTTTCACGTAC

tho5 i1 lariat	GTTCCTTACCTTTTTTTTTTTTTTTTTTC
tho5 i1 lariat (used for cDNA synthesis)	AAAAAAAAAAAAGGTAAGGAACTTTC
rap1 i2 lariat	CAATTAATGGTAAGCAAGATCATAC
rap1 i2 lariat	GCTTACCATTAATTGTTTTTTATTTTTTTTC
rap1 i2 lariat (used for cDNA synthesis)	GAAAAAAAATAAAAAACAATTAATGGTAAGC

References

1. Stepankiw N., Raghavan M., Fogarty E.A., Grimson A., Pleiss J. A. Widespread alternative and aberrant splicing revealed by lariat sequencing. *Nucleic Acids Res.* 2015; **43**:8488–8501.

2. Gruber A.R., Lorenz R., Bernhart S.H., Neuböck R., Hofacker I.L. The Vienna RNA Websuite. *Nucleic Acids Res.* 2008; **36**:W70–W74.

3. Thakran P., Pandit P.A., Datta S., Kolathur K.K., Pleiss J.A., Mishra S.K. Sde2 is an intron-specific pre-mRNA splicing regulator activated by ubiquitin-like processing. *EMBO J.* 2018; **37**:89–101.

4. Fica S.M., Oubridge C., Wilkinson M.E., Newman A.J., Nagai,K. A human postcatalytic spliceosome structure reveals essential roles of metazoan factors for exon ligation. *Science.* 2019; **363**:710–714.

5. Bergfort A., Preußner M., Kuropka B., Ilik İ.A., Hilal T., Weber G., Freund C., Aktaş T., Heyd F., Wahl M.C. A multi-factor trafficking site on the spliceosomal remodeling enzyme, BRR2, recruits C9ORF78 to regulate alternative splicing. *Nat. Commun.* 2022; **13**:1132..

6. Wang J., Tadeo X., Hou H., Andrews S., Moresco J.J., Yates J.R., Nagy P.L., Jia S. Tls1 regulates splicing of shelterin components to control telomeric heterochromatin assembly and telomere length. *Nucleic Acids Res.* 2014; **42**:11419–11432.
